# A novel seed plants gene regulates oxidative stress tolerance in *Arabidopsis thaliana*

**DOI:** 10.1007/s00018-019-03202-5

**Published:** 2019-06-27

**Authors:** Neerakkal Sujeeth, Nikolay Mehterov, Saurabh Gupta, Muhammad K. Qureshi, Axel Fischer, Sebastian Proost, M. Amin Omidbakhshfard, Toshihiro Obata, Maria Benina, Nikola Staykov, Salma Balazadeh, Dirk Walther, Alisdair R. Fernie, Bernd Mueller-Roeber, Jacques Hille, Tsanko S. Gechev

**Affiliations:** 1 BioAtlantis Ltd, Clash Industrial Estate, Tralee, Co. Kerry V92 RWV5 Ireland; 2 Center of Plant Systems Biology and Biotechnology, 139 Ruski Blvd, 4000 Plovdiv, Bulgaria; 3 Institute of Biochemistry and Biology, University of Potsdam, Karl Liebknecht Str., 24-25, 14476 Potsdam-Golm, Germany; 4 Department of Plant Breeding & Genetics, Faculty of Agricultural Sciences & Technology, Bahauddin Zakariya University, Bosan Road, Multan, 60800 Punjab, Pakistan; 5 Max Planck Institute of Molecular Plant Physiology, Am Mühlenberg 1, 14476 Potsdam-Golm, Germany; 6 Department of Molecular Pharmacology, University of Groningen, Antonius Deusinglaan 1, 9713 AV Groningen, The Netherlands; 7 Department of Plant Physiology and Molecular Biology, University of Plovdiv, 24 Tsar Assen Str, 4000 Plovdiv, Bulgaria

**Keywords:** Abiotic stress, Oxidative stress, Programmed cell death, Reactive oxygen species

## Abstract

Oxidative stress can lead to plant growth retardation, yield loss, and death. The *atr7* mutant of *Arabidopsis thaliana* exhibits pronounced tolerance to oxidative stress. Using positional cloning, confirmed by knockout and RNA interference (RNAi) lines, we identified the *atr7* mutation and revealed that *ATR7* is a previously uncharacterized gene with orthologs in other seed plants but with no homology to genes in lower plants, fungi or animals. Expression of *ATR7*-*GFP* fusion shows that ATR7 is a nuclear-localized protein. RNA-seq analysis reveals that transcript levels of genes encoding abiotic- and oxidative stress-related transcription factors (*DREB19, HSFA2, ZAT10*), chromatin remodelers (*CHR34*), and unknown or uncharacterized proteins (*AT5G59390, AT1G30170, AT1G21520*) are elevated in *atr7*. This indicates that *atr7* is primed for an upcoming oxidative stress via pathways involving genes of unknown functions. Collectively, the data reveal *ATR7* as a novel seed plants-specific nuclear regulator of oxidative stress response.

**Electronic supplementary material:**

The online version of this article (10.1007/s00018-019-03202-5) contains supplementary material, which is available to authorized users.

## Introduction

Increased levels of reactive oxygen species (ROS) in plants are a consequence of various adverse abiotic conditions such as drought, salinity, extreme temperatures, and pollutants, as well as biotic interactions that trigger the hypersensitive response (HR) to pathogens or programmed cell death (PCD) [1, 2]. An elaborate antioxidant system protects plants from ROS toxicity. In addition to their toxic nature, ROS are important signals that modulate plant growth, developmental programs, and responses to the environment [2]. ROS-induced PCD is essential for processes, such as embryo development, maturation of tracheal elements, formation of leaf shape, and leaf senescence.

Transcription factors (TFs) and regulators are induced under various stresses [3–5]. Some of them, such as the heat-inducible HSFA2 or the dehydration-responsive element binding protein (DREB)/C-repeat binding factor (CBF), activate other stress-responsive genes to confer tolerance to single or multiple stresses such as heat, drought, salt, cold, or oxidative stress [3–8]. However, our knowledge concerning the intricate ROS network that modulates stress responses, development, and cell death remains limited. Isolation and characterization of mutants with enhanced tolerance to ROS-induced PCD provide a direct way to identify components of the ROS network.

The oxidative stress-tolerant mutant *atr7*, previously obtained by chemical mutagenesis from its genetic background *loh2* (*Arabidopsis thaliana* ecotype Wassilewskija), displays high tolerance to several ROS-inducing agents such as paraquat (PQ), the catalase inhibitor aminotriazole (AT), and the fungal AAL toxin [9]. PQ is mainly active in chloroplasts, where it generates superoxide radicals by transferring electrons from photosystem I/ferredoxin to molecular oxygen; the superoxide radicals are then quickly converted to hydrogen peroxide (H_2_O_2_) by the action of superoxide dismutases [10]. AT is a potent inhibitor of catalases, the main H_2_O_2_-detoxifying enzymes in plants, and inhibiting catalase activity leads to PCD similar to the cell death observed in catalase RNAi plants [11, 12]. The *loh2* mutant, which is the genetic background of *atr7*, was obtained by knocking out a gene involved in ceramide synthesis [13] and was used to obtain the *atr7* mutant by chemical mutagenesis [9]. *Loh2* has the same phenotype as the wild-type, *A. thaliana* ecotype Wassilewskija, under normal conditions and displays the same sensitivity to PQ- and AT-induced oxidative stress as the *A. thaliana* ecotype Wassilewskija. For our study on *atr7*, we chose PQ as the ROS inducing agent. Here, we identify *ATR7* by map-based cloning and show that it encodes a novel nuclear-localized protein with a previously unreported function. The gene is specific to seed plants; there are no homologs in lower plants (algae, ferns, lycopods, and mosses), fungi, and animals. Molecular analyses of the *atr7* transcriptome (RNA-seq) and metabolome (GC–MS) identified genes and pathways that are highly up- and downregulated in *atr7*. Their potential role in the oxidative stress response is discussed.

## Results

### The *atr7* mutant tolerates PQ-induced oxidative stress

**Fig. 1:**
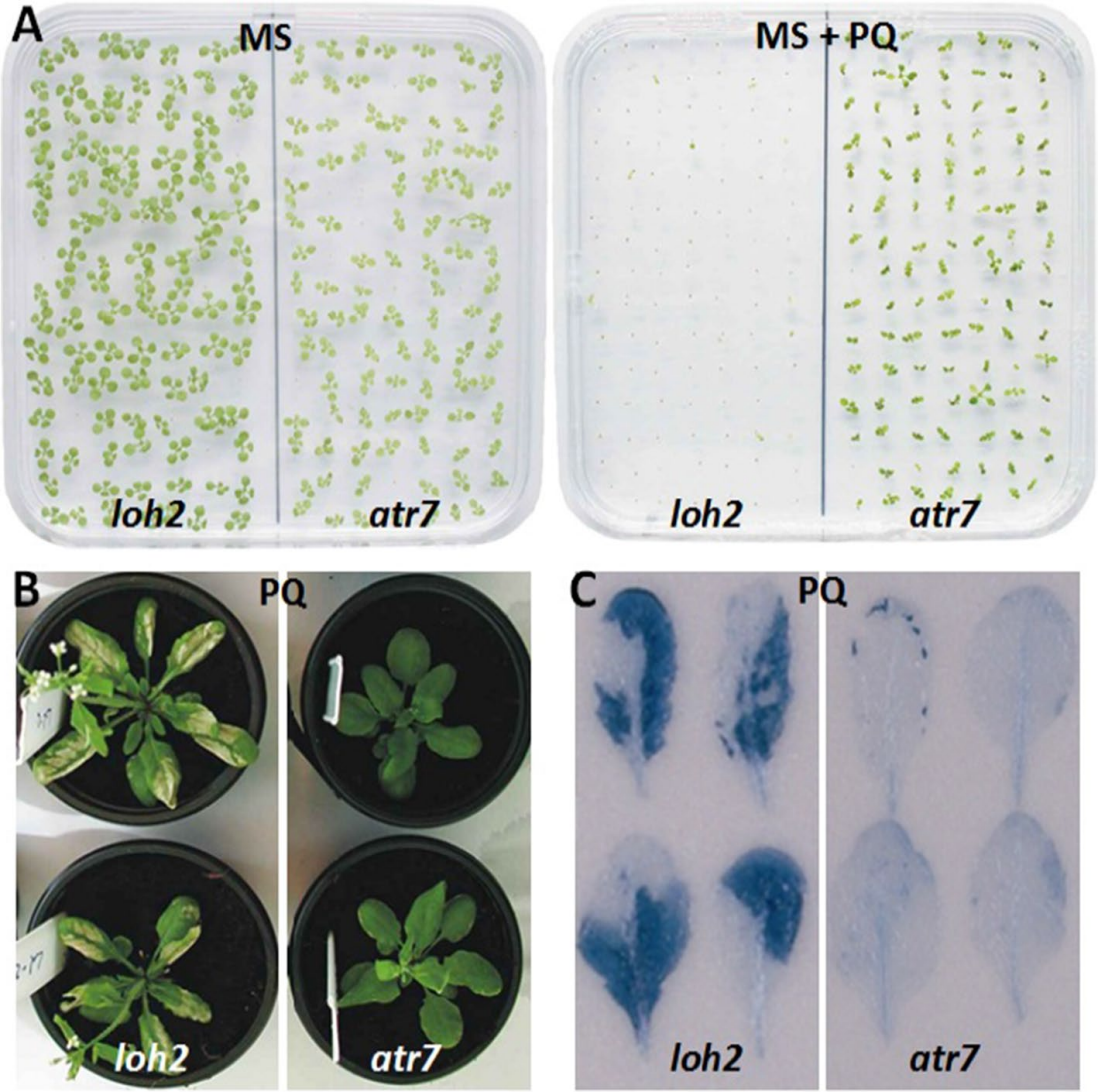
*Atr7* exhibits enhanced tolerance to oxidative stress. **a** 1-week-old *loh2* and *atr7* seedlings on Murashige and Skoog (MS) plant growth media (left) or MS media supplemented with 1 μM paraquat (PQ) (right). **b** Mature *loh2* and *atr7* plants grown in soil, sprayed with 15 μM PQ. **c** Rosette leaves from *loh2* and *atr7* plants sprayed with 15 μM PQ and stained with trypan blue to detect cell death

When plants were grown on Murashige and Skoog (MS) media containing PQ, *loh2* seedlings bleached and died while *atr7* seedlings stayed green and alive (Fig. 1a), as previously reported [9]. When grown in soil and sprayed with PQ at the rosette stage, *loh2* plants developed massive necrotic lesions while *atr7* lacked cell death symptoms (Fig. 1b). Trypan blue staining confirmed massive necrosis in *loh2* and the absence of cell death in *atr7* (Fig. 1c).

Treatment with PQ elevated the endogenous ROS levels, as assessed by staining with diaminobenzidine (detecting hydrogen peroxide) and gene expression analysis of two ROS marker genes (Supplementary Fig. 1). The increase in ROS was much more prominent in *loh2* but also evident in *atr7*. Furthermore, *atr7* had higher basal levels in the absence of stress, as compared with unstressed *loh2* plants (Supplementary Fig. 1).

### Molecular cloning of *ATR7* by map-based approach

**Fig. 2:**
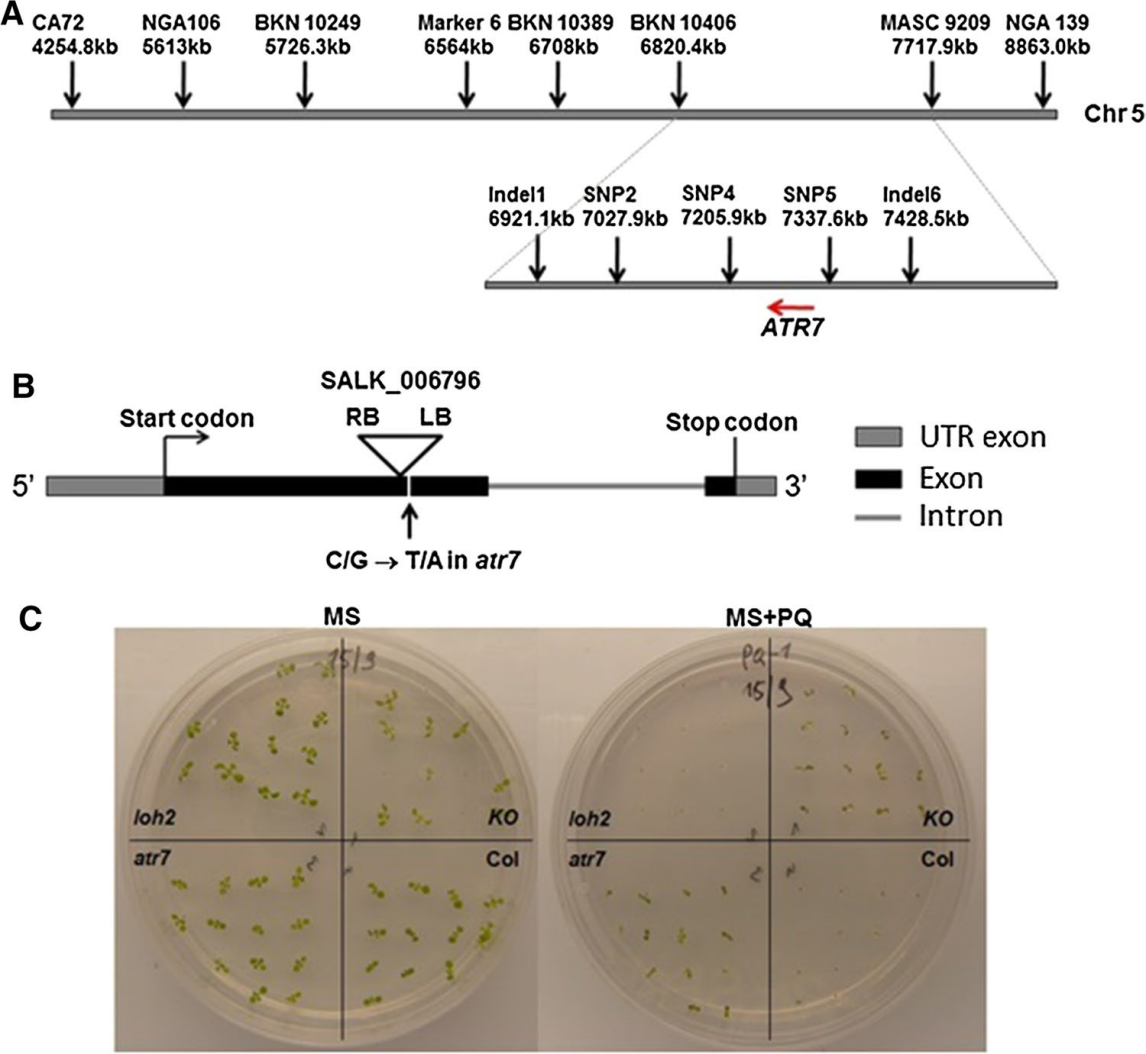
Identification of *ATR7* by molecular cloning. **a** Genetic mapping of the *atr7* mutation. Markers used for the fine mapping and their distance on chromosome 5 are presented above. The relative position of *ATR7* between SNP4 and SNP5 is shown below. The red arrow indicates the direction of *ATR7* transcription. **b** Genomic structure of *ATR7* (AT5G21280) with the position of the *atr7* point mutation (G → A) and the location of the T-DNA insertion in *ATR7* in the knockout line SALK_006796 indicated (RB and LB are the right and left borders, respectively, of the T-DNA). **c** 1-week-old seedlings of *loh2, atr7*, *atr7* knockout (SALK_006796), and wild-type Columbia-0 (Col) grown on Murashige-Skoog (MS) medium (left) or MS medium supplemented with 1 μM paraquat (MS + PQ, right). The *loh2* and the wild-type Columbia-0 plants die on media with PQ (100% mortality), whereas all *atr7* mutant and *atr7* knockout (SALK_006796) plants survive on PQ (100% survival)

To identify the mutation responsible for the tolerance to oxidative stress, *atr7* plants were crossed with *A. thaliana* ecotype Columbia-0. All F_1_ seedlings examined showed sensitivity to 1.5 µM PQ, similar to wild-type (WT) plants. The F_2_ population of 2909 individuals segregated in a 3 (susceptible): 1 (tolerant) Mendelian fashion, indicating that *atr7* is a recessive mutation at a single nuclear locus. Coarse mapping with 50 PQ-tolerant plants located *atr7* between the SSLP markers CA72 and NGA139 on chromosome 5 (Fig. 2a). Further fine mapping with a larger population of 604 individuals delimited the *atr7* locus within a region of approximately 100 kb (Fig. 2a). Sequencing of the candidate genes in this region using the Illumina technology revealed a point mutation (C/G to T/A transition) in the first exon of gene *AT5G21280*, resulting in a premature stop codon (Fig. 2b, Supplementary Fig. 2a). Screening of the TAIR database (http://www.arabidopsis.org/) identified a knockout line (KO, SALK_006796) with a T-DNA insertion in the first exon of *AT5G21280* (Fig. 2b). Homozygous KO plants were tolerant to PQ, like the originally isolated *atr7* mutant (Fig. 2c). The *ATR7* KO mutant does not have any obvious phenotype in the absence of stress with plant growth and fertility being normal. End-point RT-PCR with primers recognizing the ends of the *ATR7* coding sequence confirmed the absence of full-length transcript in both *atr7* and *ATR7* KO (Supplementary Fig. 3). The lack of *ATR7* expression in both the *atr7* point mutant and the *ATR7 KO* mutant was verified by qRT-PCR with primers upstream of the T-DNA insertion. Additionally, we inhibited *ATR7* expression by generating RNAi lines in both *loh2* and the Wassilewskija background (Supplementary Fig. 2b). The resulting RNAi lines were as tolerant to PQ-induced oxidative stress as the *atr7* mutant (Supplementary Fig. 2b). Finally, a complementation line expressing the wild-type *AT5G21280* gene in the *atr7* background showed sensitivity to PQ similar to that of wild-type plants (Supplementary Fig. 2c). Taken together, these results demonstrate that *ATR7* is *AT5G21280*.

### *ATR7* encodes a novel nuclear-localized protein with unknown function

*ATR7* encodes a previously uncharacterized protein of unknown function. The ATR7 protein has a length of 302 amino acids (Supplementary Fig. 4), a calculated molecular weight of 33,608.61 Da, and an estimated isoelectric point of 5.14. ATR7 has some similarity with hydroxyproline-rich glycoproteins (HRGPs), but unlike typical HRGPs exported to the cell wall via a signal peptide, ATR7 protein lacks a signal peptide and its hydropathy plot predicts the absence of trans-membrane domains. A multiple sequence alignment shows few stretches of conserved sequences, the longest one is shown in Supplementary Fig. 4. A Pfam search for that sequence did not identify any known motif/domain. Interestingly, however, a significant portion of the ATR7 protein is predicted to be intrinsically disordered in the Database of Disordered Protein Predictions (D^2^P^2^; http://d2p2.pro/).

**Fig. 3:**
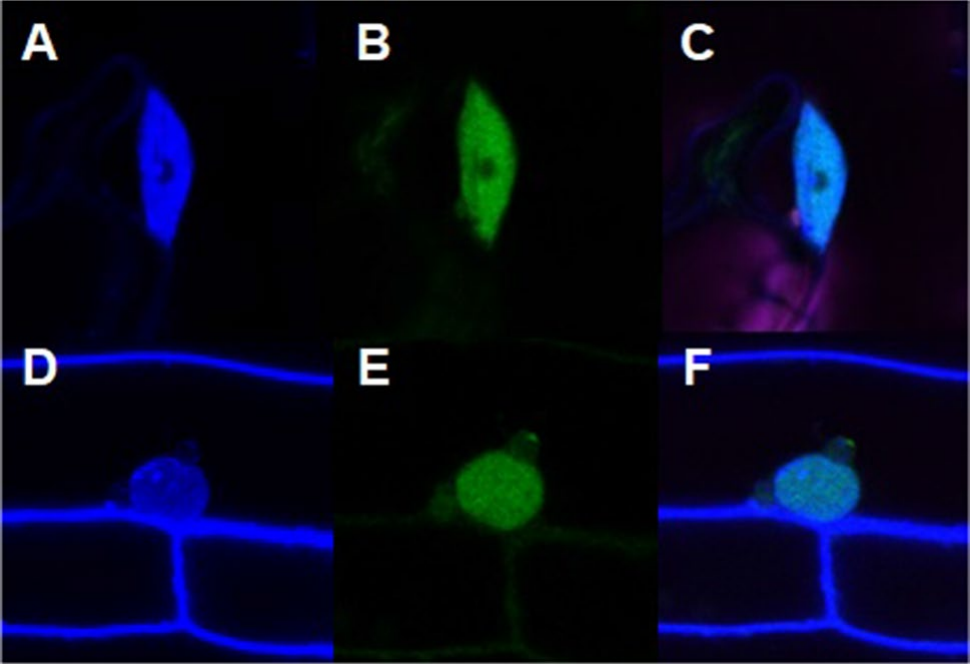
Nuclear localization of ATR7. GFP signal is detected in nuclei of cells from leaves and roots of Arabidopsis plants stably transformed with the *ATR7*-*GFP* construct. Nuclei are counterstained with 4′,6-diamidino-2-phenylindole (DAPI). **a** DAPI-stained nucleus in the leaf. **b** GFP signal in the nucleus of the leaf. **c** Merged image. **d** DAPI-stained nucleus in the root. **e** GFP signal in the nucleus of the root. **f** Merged image

The subcellular localization database for Arabidopsis proteins (SUBA3, http://suba.plantenergy.uwa.edu.au/) predicts nuclear localization of ATR7, which we confirmed by expressing an ATR7-GFP fusion from the Cauliflower Mosaic Virus (CaMV) *35S* promoter in stably transformed transgenic plants (Fig. 3). The overexpression of the ATR7-GFP protein or the ATR7 protein alone did not result in any visible phenotype under optimal growth conditions or under PQ-induced oxidative stress.

**Fig. 4:**
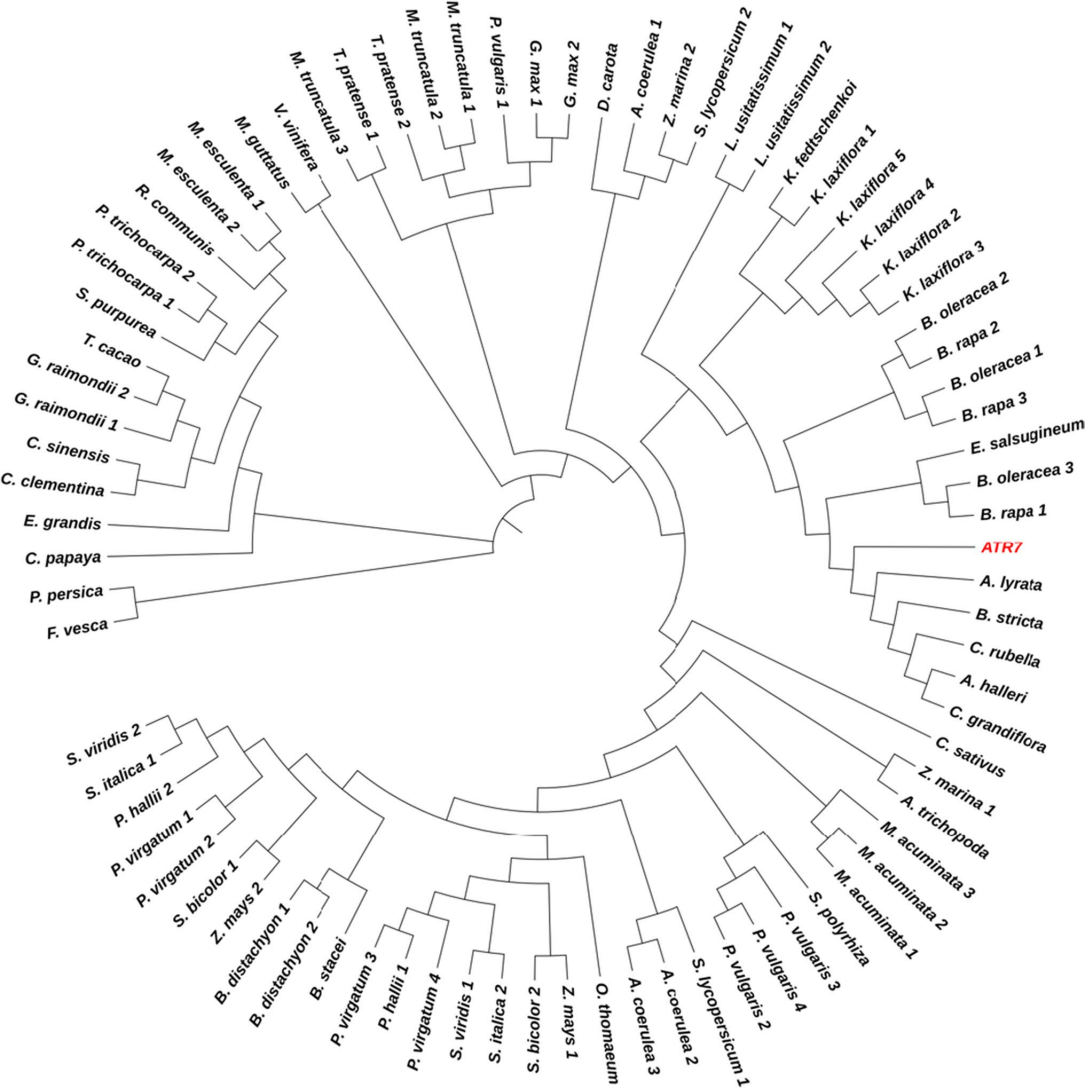
Phylogenetic tree of *ATR7* and 80 orthologs from plants with sequenced genomes, available at Phytozome. Multiple sequence alignment of protein sequences was done using MUSCLE. The tree was inferred employing the neighbor-joining method using simple phylogeny after distance correction. *ATR7* is shown in red. Description of the sequences is provided in Supplementary Table 1

According to the PLAZA 4.0 database for comparative genomics (http://bioinformatics.psb.ugent.be/plaza/) [14], *ATR7* homologs are present in most higher plant genomes. We extended the homology study further to cover additional plant genomes available to date and prepared a phylogenetic tree (Fig. 4). A comprehensive list of all *ATR7* homologs is presented in Supplementary Table 1. Homologs are present in monocot and dicot crops such as rice, cucumber, cabbage, strawberry, and grapevine. Interestingly, however, no homologs of *ATR7* were found in animals or fungi, and no genes with significant sequence similarities were found in lower plants (algae, mosses, ferns, and lycopods). However, there is a single match with *Amborella trichopoda*, a species believed to be one of the earliest angiosperm plants. A multiple sequence alignment (Supplementary Fig. 4) shows that ATR7 is more closely related to its homologs from the Brassicaceae family than to species from other families. Collectively, our observations indicate recent evolution of the *ATR7* gene in flowering plants.

**Table 1:** Expression patterns of *ATR7* and genes encoding key abiotic and oxidative stress-related nuclear-localized proteins. The expression at normal growth conditions and under paraquat (PQ)-induced oxidative stress is given as TMM values. Data are means of three replicates ± SD

Gene name	*loh2*	*loh2* PQ	*atr7*	*atr7* PQ
*ATR7*	11.28 ± 1.7	72.8 ± 6.75	6.95 ± 0.65	13.62 ± 0.76
*HSFA2*	0.86 ± 0.91	6.85 ± 2.34	4.76 ± 4.6	5.94 ± 8.7
*DREB19*	0.55 ± 0.11	47.76 ± 2.34	5.68 ± 1.77	18.79 ± 1.15
*ZAT10*	3.75 ± 1.44	23.53 ± 0.64	16.23 ± 6.95	14.46 ± 2.76
*ZAT12*	7.33 ± 1.73	86.66 ± 14.1	34.99 ± 10.9	84.89 ± 4.26
*CHR34*	0.07 ± 0.02	8.14 ± 1.23	11.6 ± 1.48	26.92 ± 14.5
*ANAC085*	0.12 ± 0.11	3.98 ± 0.45	4.01 ± 1.98	3.93 ± 0.94
*AT1G21520*	3.45 ± 0.73	69.66 ± 7.65	170.21 ± 39.3	303.21 ± 92.3

### *ATR7* gene expression is induced under oxidative and abiotic stresses

Despite the fact that a mutation of *ATR7* has such a striking effect on oxidative stress tolerance and cell death responses in *A. thaliana*, little is known about its molecular mode of action and its environmentally affected pattern of expression. To fill this gap, we analyzed RNA-seq data to determine how the *ATR7* gene is expressed during development, in different organs/tissues, or during abiotic and oxidative stresses. According to the RNA-seq data available through Araport (https://www.araport.org/), *ATR7* is expressed in all plant organs. However, the RNA-seq data were scarce, different experiments were not comparable with one another, and crucial information about *ATR7* expression under stress was missing. Thus, we generated our own data by performing qRT-PCR expression profiling with plants subjected to oxidative (hydrogen peroxide) and abiotic stresses (heat, cold, CdCl_2_, and osmotic stress induced by mannitol). According to our data, *ATR7* was notably induced by hydrogen peroxide, salt stress, CdCl_2_, and mannitol but not by sub-optimal temperatures such as cold or heat stress (Supplementary Fig. 5).

### Transcriptome analysis reveals activation of both known stress protection genes and a set of novel genes in *atr7* that collectively contribute to oxidative stress tolerance

**Fig. 5:**
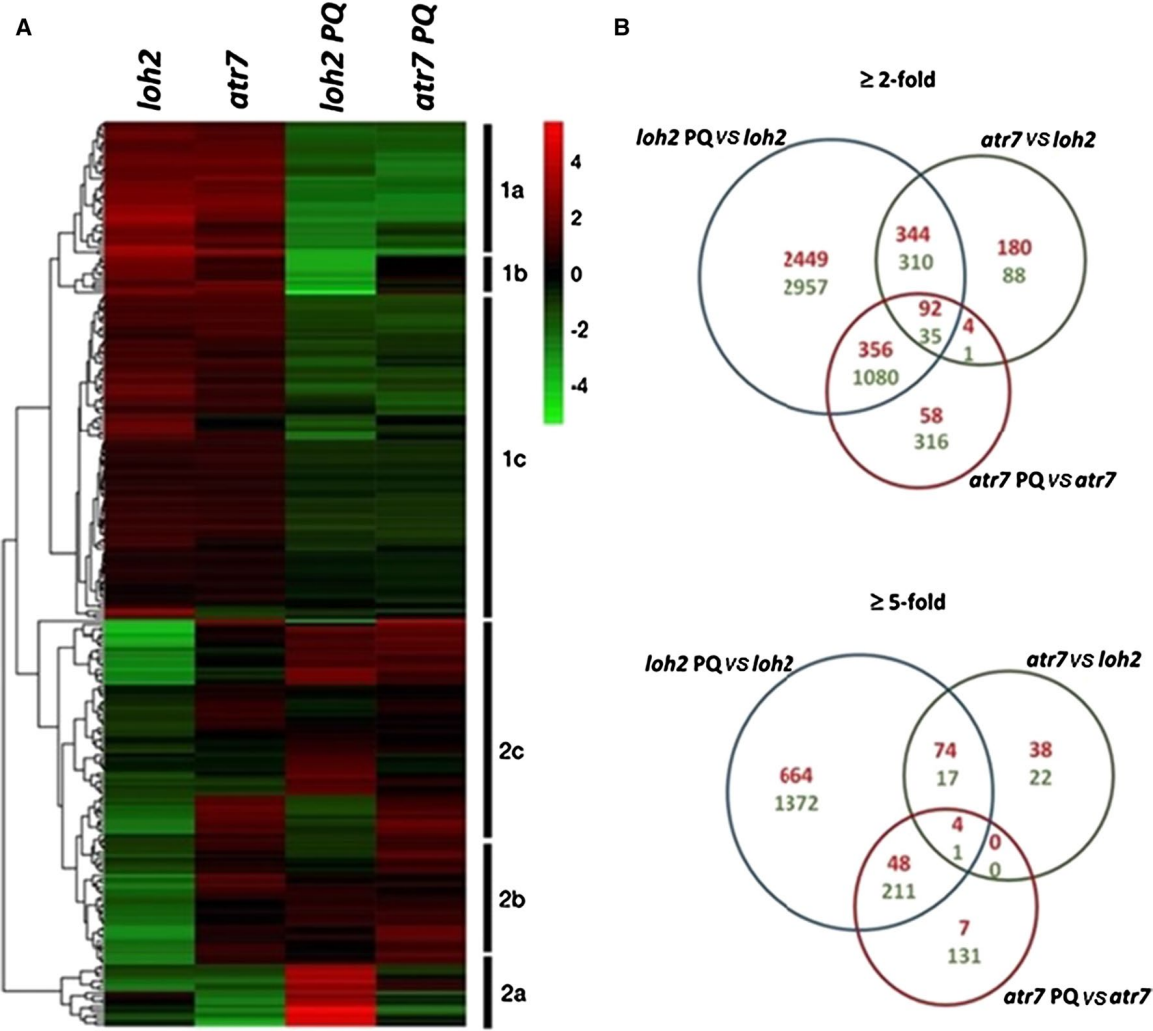
Transcriptome re-programming due to paraquat-induced oxidative stress in *loh2* and *atr*7. **a** Hierarchical linkage clustering of 263 genes representing the 100 most up- or downregulated genes after paraquat (PQ) treatment in the two genotypes, as well as the 100 most regulated genes (induced or repressed) between *loh2* and *atr7* grown under normal conditions (without stress). *Atr7* and its genetic background *loh2* were grown on Murashige and Skoog (MS) medium without PQ (unstressed controls) or with 1 μM PQ (oxidative stress), and gene expression was determined by RNA-seq. Each row represents the expression profile of an individual gene, given as mean subtracted average log2 gene expression values. Red color indicates upregulation while green indicates downregulation. Data are log2 normalized mean-centered TMM values. **b** Venn diagram of genes altered in expression in *atr7* in the absence of stress (*atr7 vs*. *loh2*, green circles), as well as genes regulated by paraquat in *loh2* (*loh2* PQ *vs*. *loh2*, blue circle) and *atr7* (*atr7* PQ *vs*. *atr7*, red circle). Genes induced by at least 2- or 5-fold are given in red numbers, while genes repressed by at least 2- or 5-fold are in green numbers

To better understand *ATR7*´s mode of action, *atr7* and its genetic background *loh2* were grown under standard condition or subjected to PQ-induced oxidative stress, and RNA was isolated for transcriptome analysis by RNA sequencing (RNA-seq). PQ-induced oxidative stress caused significant transcriptional re-programming in both *loh2* and *atr7*, with more prominent changes in *loh2*. Gene expression levels are given in Supplementary Table 2. The RNA-seq data were verified by qRT-PCR analysis of eight randomly selected genes. In the absence of stress, expression of 1054, 156, and 72 genes was at least 2-, 5- and 10-fold, respectively, altered (up or down) in *atr7* compared to *loh2*. Importantly, while oxidative stress altered the expression of 7623, 2391, and 1029 genes by at least 2-, 5- and 10-fold in *loh2*, only 1942, 402, and 157 genes, respectively, were affected in *atr7*. The 100 genes that were most regulated (up or down) by PQ in *loh2* and in *atr7*, as well as the 100 most regulated (up or down) genes in the *atr7* mutant versus *loh2* in the absence of stress, were used to generate Fig. 5a. Figure 5b presents an overlap of the genes regulated by more than 2- or 5-fold in the three comparisons (*loh2* PQ *vs*. *loh2*, *atr7* PQ *vs*. *atr7*, and *atr7 vs*. *loh2*).

Analysis of the differentially expressed genes with the largest fold change (up- and downregulated) represented in Fig. 5a revealed two distinct clusters. Cluster 1 contains genes with a similar expression level in *loh2* and *atr7* in the absence of stress (Fig. 5a). The genes in cluster 1A are repressed in both genetic backgrounds. It is enriched in genes encoding proteins related to cell wall expansion, growth, and redox processes, such as arabinogalactans, xyloglucan endotransglycosylases, various mono- and dioxygenases, oxidases, and cytochrome P450 proteins (Fig. 5a, Supplementary Table 3). Cluster 1B is enriched in genes encoding proteins involved in photosynthesis and carbon assimilation, such as RuBisCO small subunit, chlorophyll a/b binding protein 2, LHCII subunit B, and carbonic anhydrase (Fig. 5a). This cluster contains genes mainly downregulated in *loh2*, but not in *atr7*. This clearly shows that photosynthesis is inhibited in stress-sensitive *loh2* but not in stress-tolerant *atr7*.

Cluster 2 contains genes with expression patterns that are rather different between *loh2* and *atr7*, even in the absence of stress. A small group of genes was highly upregulated by PQ treatment in *loh2*, but not in *atr7* (Fig. 5a, cluster 2A). Indeed, some of these genes were downregulated in *atr7* under normal growth conditions and further repressed in the mutant exposed to PQ (Fig. 5a, cluster 2A). Representatives of this group are genes encoding dihydroneopterin aldolase, UDP-glucosyl transferase 76E12, and a glycosyl hydrolase. Sub-clusters 2B and 2C contain genes whose expression levels in unstressed plants were higher in *atr7* than in *loh2* (Fig. 5a). These clusters are enriched in plant-specific genes, several of which encode nuclear-localized proteins (enzymes, TFs, chromatin modifiers, etc.). Notably, a significant percentage (16%) of those genes has not been functionally characterized so far. The genes selected for clustering contained NAC transcription factors and heat shock proteins all of which are included in cluster 2C (Fig. 4a). Supplementary Table 3 contains the genes most regulated in *atr7* in the absence of stress. Gene ontology (GO) enrichment analysis indicated that the two categories with the most upregulated genes in the *atr7* mutant are “response to heat” and “rRNA processing”, while the category with the most downregulated genes is “photosynthesis” (Supplementary Fig. 6). Genes encoding key abiotic and oxidative stress-related transcription factors (*DREB19*, *HSFA2*, *ZAT10*, *ZAT12*) [9], chromatin remodelers (*CHR34*), and many genes encoding uncharacterized proteins (*ANAC085*, *AT5G59390*, *AT1G30170*, *AT1G21520*), were constantly upregulated in *atr7* in the absence of stress (Table 1, Supplementary Table 3). Among the proteins encoded by the 100 most upregulated genes, 32% are predicted to be localized in the nucleus based on GO annotations. Combined with the nuclear localization of ATR7 protein, the two observations collectively suggest a functional role of ATR7 in the nucleus. It is likely that these key abiotic stress genes upregulated in *atr7* jointly contribute to its abiotic and oxidative stress tolerance. DREB19, HSFA2, ZAT10, and ZAT12 have well-established roles in abiotic and oxidative stress responses, playing important roles in tolerance to drought, salinity, and high temperature, as well as for “thermomemory” [4–6, 15].

Pathway analysis of the gene expression data using MapMan revealed an upregulation of many abiotic stress-related genes in *atr7*, as well as of genes from the ubiquitin- and autophagy-dependent degradation pathways (Supplementary Fig. 7). Genes from the mitochondrial electron transport chain were also upregulated in *atr7* (Supplementary Fig. 7). On the other hand, genes related to biotic stress, jasmonic acid signalling, chlorophyll biosynthesis, photosynthesis (light reactions, Calvin cycle), and photorespiration were downregulated in *atr7* (Supplementary Fig. 7).

To identify genes co-expressed with *ATR7*, we used the CoNekT platform which uses > 900 *A. thaliana* samples to build a co-expression network for all genes. The cluster corresponding to *ATR7* contains 60 genes (Supplementary Table 4). GO enrichment analysis of these genes showed enrichment of terms such as “response to hypoxia”, “signal transduction”, “regulation of ROS metabolic process”, “seed germination”, “lipid storage”, and “response to abiotic stimulus”. Among these genes, 33 were also induced (at least twofold) in *loh2* upon PQ stress (Supplementary Table 4).

### Metabolome reconfigurations contribute to the oxidative stress tolerance in *atr7*

**Fig. 6:**
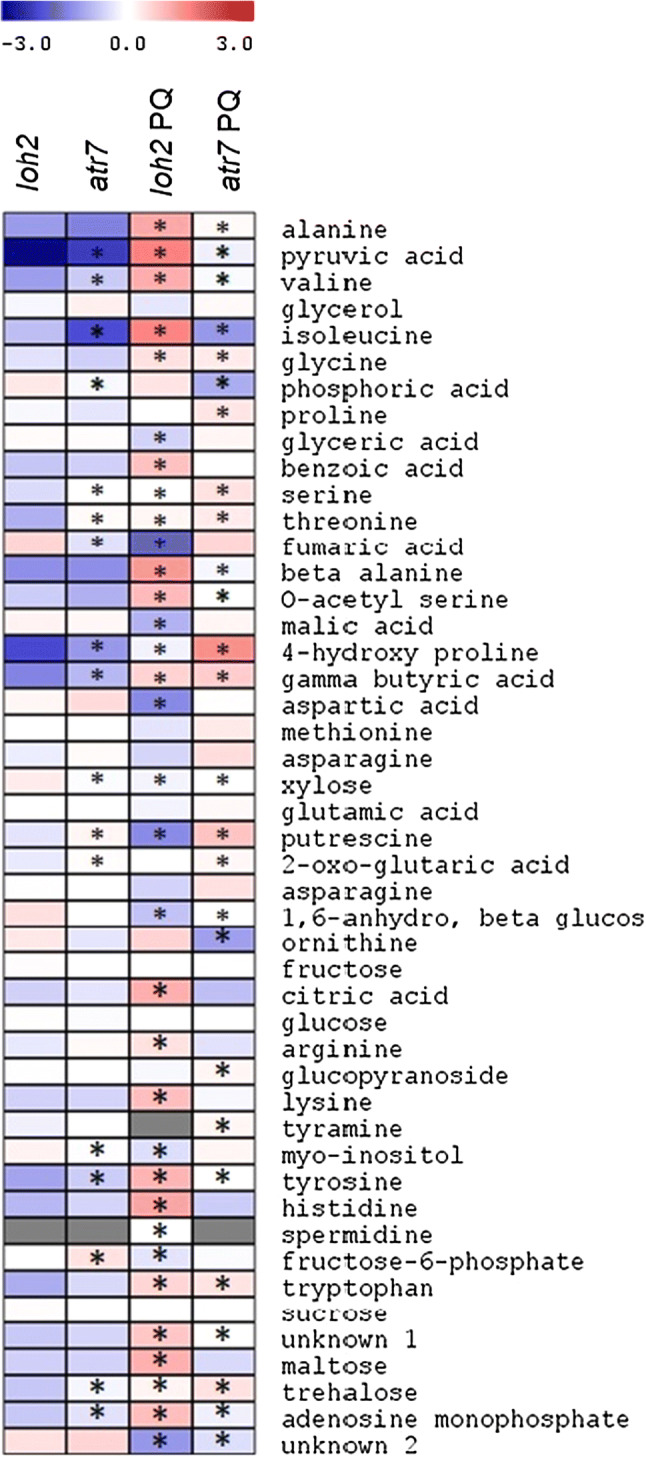
Relative levels of primary metabolites in *loh2* and *atr7* grown under normal conditions or under paraquat-induced oxidative stress (PQ). Red and blue depict increases and decreases, respectively, in content. Asterisks indicate values that are statistically different from the *loh2* levels (Student’s *t* test, *p *< 0.05). The data are means of six biological replicates

To extend our knowledge about the molecular processes occurring during oxidative stress, we conducted metabolite profiling of *loh2* and *atr*7 plants during PQ-induced oxidative stress. Relative metabolite levels in *loh2* and *atr*7 plants grown in non-stress conditions and under PQ-induced oxidative stress are presented in Fig. 6 and Supplementary Table 5. The global picture reveals that, with a few exceptions, the metabolite profiles of *loh2* and *atr7* are very similar in the absence of stress. Principal component analysis confirms this (Supplementary Fig. 8). Upon PQ-induced oxidative stress, however, there are a number of changes in *atr7*, but the most dramatic changes occur in *loh2*. More specifically, the levels of many amino acids, such as alanine, valine, tyrosine, isoleucine, and lysine, were significantly elevated in *loh2*, compared with unstressed controls and with PQ-treated *atr7*. A similar pattern was observed for a number of other metabolites, including the organic acids pyruvic, citric, and benzoic acid. At the same time, other metabolites such as aspartic acid, fumaric acid, malic acid, and putrescine, significantly decreased in *loh2* upon PQ treatment, but not in *atr7*. Moreover, putrescine and proline, two metabolites with prominent stress-protective roles, showed even higher levels in PQ-treated *atr7* than in both *loh2* and unstressed controls.

### The *atr7* mutant is less sensitive to drought stress

The induction of many stress-related TFs in the *atr7* mutant, as well as the induction of the *ATR7* gene itself by oxidative and abiotic stresses, suggested that the *atr7* mutant may be altered in abiotic stress responses. We evaluated the response of *atr7* and *loh2* to drought stress. Under the conditions used, *loh2* showed cessation of growth, visible leaf damage, significant wilting (loss of water, as recorded by the decreased relative water content), and increased electrolyte leakage (Supplementary Fig. 9). Compared with *loh2, atr7* showed much less visible damage of the leaves, less growth retardation, preserved relative water content, and showed little increase in ion conductivity (Supplementary Fig. 9).

## Discussion

Previously, we showed that the *atr7* mutant is more tolerant to oxidative stress induced by PQ and AT than *loh2* [9]. Under normal growth conditions, *atr7* looks a bit smaller than *loh2* and its chlorophyll level is 90% of that of *loh2* [9]. Treatment with PQ resulted in a complete loss of chlorophyll in *loh2* and 100% plant mortality, while the chlorophyll level of *atr7* was fully retained and all *atr7* plants remained viable. Furthermore, using a home-made platform for qRT-PCR analysis, we demonstrated that several ROS marker genes and transcription factors are upregulated in *loh2* under oxidative stress and these genes have higher basal expression in *atr7* compared with *loh2* in the absence of stress [9]. These preliminary results suggested a possible link with abiotic stress, as some of the transcription factor genes were known to be involved in abiotic stress responses.

Here, we show that the *atr7* mutant, which has a striking oxidative stress-tolerant phenotype, carries a mutation in locus *AT5G21280*. We also show that the *ATR7* gene encodes a novel nuclear-localized protein of unknown function. We identified *ATR7* homologs in many flowering plants, including important crops, but no homologs were identified in lower plants such as algae, mosses, ferns, and lycopods. No studies on the biological role of the *ATR7* gene or its protein have been published to date and none of the *ATR7* homologs in other species have been functionally characterized. Furthermore, there are no known protein domains present that can be used to infer a putative function of the proteins.

While the *atr7* mutant is more tolerant to both PQ and AT than *loh2*, other oxidative stress-tolerant mutants, such as *par1*, *par2, pdr11,* or *oxt1*, are more specific to either PQ or AT, respectively [10, 16–18]. *Par2* enhances PQ tolerance by elevating nitric oxide (NO) levels, while *pdr11* and *par1* are blocked in transport of PQ into the cell or chloroplasts, respectively. *PAR2* encodes an *S*-nitrozoglutathione reductase (GSNOR) involved in the metabolism of *S*-nitrosoglutathione (GSNO), a major bioactive donor of NO [10]. The increased NO levels in *par2* upregulate the defense responses against PQ [10]. *PDR11* (*AT1G66950*) encodes an ABC transporter localized in the plasmalemma, while *PAR1* encodes an L-type amino acid transporter or amino acid permease localized in the Golgi apparatus [16, 17]. Thus, the two mutants show high levels of tolerance to PQ due to the inability to transport it into the cytoplasm and chloroplasts, but unlike *atr7* they are not tolerant to other ROS inducing agents. Another PQ-tolerant mutant, *pqt3*, is defective in an E3 ubiquitin-protein ligase, which is a negative regulator of histone methylase PRMT4b [19]. PRMT4b itself can activate gene expression of the antioxidant genes *ASCORBATE PEROXIDASE 1* (*APX1*) and *GLUTATHIONE PEROXIDASE 1* (*GPX1*), thus alleviating oxidative stress. Two other mutants, *oxt1* and *oxt6*, have been isolated as being more tolerant to AT. The *oxt1* mutant is disrupted in a gene encoding ADENINE PHOSPHORIBOSYLTRANSFERASE (*APT1*), an enzyme that converts adenine to adenosine monophosphate (AMP), and accordingly o*xt1* plants have elevated levels of adenine as well as elevated APX enzyme activities [18]. The *oxt6* mutation results in the inactivation of a gene that encodes the 30-kD subunit of THE CLEAVAGE AND POLYADENYLATION SPECIFICITY FACTOR (CPSF30), which eventually results in upregulation of stress-protective genes in the *oxt6* mutant [20].

Our RNA-seq analysis revealed clear differences between *loh2* and *atr7*. Of note, many more genes were induced by PQ in *loh2* than in *atr7*. This correlated with a higher level of oxidative stress in PQ-treated *loh2*. Besides the higher number of genes affected by PQ in *loh2*, it is obvious that the number of genes repressed by PQ in both *loh2* and *atr7* is higher than the number of PQ-induced genes. Another notable observation is the large number of genes regulated in the *atr7* mutant compared with *loh2* in the absence of stress (*atr7 vs*. *loh2*, unstressed controls). While the reason for this difference in gene expression is unclear, enhanced basal levels of hydrogen peroxide in *atr7* may have contributed to the differential gene expression between *atr7* and *loh2* in the unstressed plants. The upregulated genes are much more than those downregulated (620 up-/434 downregulated by at least twofold; 116 up-/40 downregulated by at least fivefold).

The differentially expressed gene with the largest upregulation in the *atr7* mutant encodes MBS1 (METHYLENE BLUE SENSITIVITY 1), a small zinc finger protein that mediates chloroplast-to-nucleus singlet oxygen (^1^O_2_) signaling and is essential for tolerance to photo-oxidative stress [21]. *MBS1* is virtually not expressed in *loh2*, while it is well expressed both in stressed and unstressed *atr7*. The Arabidopsis loss-of-function mutant *mbs1* is hypersensitive to photo-oxidative stress, whereas overexpression of *MBS1* leads to enhanced stress tolerance [21]. MBS1 is also essential for acclimation to ^1^O_2_-induced oxidative stress and acts downstream of β-cyclocitral, a second messenger that mediates ^1^O_2_ responses [22]. Thus, upregulation of *MBS1* in *atr7* likely contributes to the primed condition against oxidative stress. The second most upregulated gene (*AT1G23410*) in *atr7* encodes ribosomal protein S27a, whose function in Arabidopsis is unknown. In addition, several other ribosomal genes were highly upregulated in *atr7* (Supplementary Table 3). GO analysis of the genes upregulated in *atr7* indicated enrichment of ribosomal RNA-processing genes, genes involved in 60S ribosome biogenesis, and genes encoding ribosomal proteins, indicating a link between *ATR7* and the ribosome (Supplementary Fig. 6).

A number of the genes with high expression in *atr7* encode proteins that have not been functionally characterized or have no known functions. Among the top 20 most upregulated genes in *atr7* in unstressed conditions, *AT5G59390*, *AT1G30170*, and *AT1G21520* encode proteins with unknown functions and unknown sub-cellular locations. Incidentally, in a study on 50 proteins with unknown functions, overexpression of *AT1G21520* in Arabidopsis was found to alleviate PQ-induced oxidative stress [23]. Thus, it is likely that *AT1G21520* together with the other known and unknown proteins mentioned above collectively contribute to the enhanced oxidative stress tolerance of *atr7*.

The *ATR7* gene is induced by a number of oxidative and abiotic stresses, including H_2_O_2_, PQ, CdCl_2_, salinity, and osmotic stress, but not by chilling and heat stress. This, together with the induction of many stress-related genes in the *atr7* mutant, suggested a possible altered response of *atr7* to abiotic stresses. Indeed, *atr7* exhibited reduced sensitivity to drought stress, further substantiating the link between oxidative stress and drought. More studies are needed in the future to determine the response of *atr7* in other abiotic stresses such as salt, heavy metals (CdCl_2_), and extreme temperatures.

Metabolome reconfigurations play important roles in the adaptation of plants to abiotic and oxidative stresses, as readjustments are needed both to maintain cellular homeostasis and to produce metabolites that can protect from stress [24]. The metabolome of PQ-stressed *loh2* is strikingly different from the metabolome of unstressed *loh2*, while the metabolome of PQ-stressed *atr7* is not so much different from that ofunstressed *atr7*. Particularly notable in this respect are the levels of stress metabolites including β-alanine, *myo*-inositol and proline as well as the amino acids that best reflect enhanced protein degradation, namely lysine and the branched chain and aromatic amino acids [25]. Putrescine and proline show the highest levels in PQ-treated *atr7*. Proline accumulation in particular is known to ameliorate several stresses [26]. Putrescine has a well-documented role in oxidative and abiotic stress tolerance, including drought stress [27]. With regard to PQ-induced oxidative stress, it has been suggested that putrescine competes with PQ for the polyamine transport system, thereby reducing the uptake of PQ [28]. This notion is supported by the discovery that natural variation in the polyamine transporter RMV1 (resistant to methyl viologen 1) determines PQ tolerance in Arabidopsis [29]. Further substantiating this link, the PQ-resistant mutant *par1*/*pqr2* encodes a defective polyamine transporter [27]. All this collectively supports the conclusion that atr7 is tolerant to oxidative stress.

In conclusion, we identify ATR7 as a novel regulator of oxidative stress tolerance in *Arabidopsis thaliana*. The fact that oxidative- and abiotic stress-responsive transcription factors are upregulated in the *atr7* mutant already in the absence of stress indicates that *atr7* is primed for an upcoming oxidative stress. ATR7 is a nuclear protein that represses expression of key oxidative- and abiotic stress-related genes. Many of the genes with altered expression in *atr7* encode proteins with unknown functions, suggesting that a previously unidentified molecular mechanism contributes to the oxidative stress tolerance in the *atr7* mutant. *ATR7* itself is a recently evolved gene, specific for seed plants and with no homologs in lower plants, fungi or animals. The enhanced tolerance of *atr7* to drought stress and the presence of *ATR7* homologs in agriculturally important species raise the possibility for crop improvement through modulation of ATR7 levels.

## Materials and methods

### Plant material, growth conditions, stress treatments, and stress assessment

The following plant material was used in this study: *Arabidopsis thaliana* ecotypes Columbia (Col-0) or Wassilewskija (Ws), *Arabidopsis thaliana loh2* and *atr7* mutants, described earlier [9, 29] and the *atr7* knockout line SALK_006796 obtained from the Nottingham Arabidopsis Stock Center (http://arabidopsis.info/).

Plants were grown either in vitro on Murashige and Skoog (MS) plant media in Percival plant growth chambers (14 h light/10 h dark period, photosynthetic photon flux density 80 μmol m^−2^ s^−1^, 22 °C), or on soil under standard greenhouse conditions (14 h light/10 h dark period, photosynthetic photon flux density 400 μmol m^−2^ s^−1^, 22 °C and relative humidity 70%). Before sowing, seeds were surface sterilized for 2 h with gaseous chlorine derived from sodium hypochlorite and hydrochloric acid in a closed glass vessel. For most of the in vitro experiments, one-week-old plants were used for different measurements (RNA isolation, extraction of metabolites), while mature plants at rosette leaves stage were used for experiments on soil.

Paraquat (PQ) was either included in MS media at a concentration of 1 or 1.5 μM, or applied by spraying at concentrations of 15 or 25 μM. The presence of dead cells was shown by lacto-phenol trypan blue staining. In brief, PQ-treated and control, *loh2* and *atr7* rosette leaves were boiled in ethanol-diluted trypan blue solution (10 mL of phenol, 20 mL of 50% glycerol, 10 mL of lactic acid, 10 mL of distilled water, and 0.02 g of trypan blue) for 2 min, followed by 1 h incubation. De-staining was performed by several washings in saturated chloral hydrate solution (1 kg of chloral hydrate dissolved in 400 mL of distilled water, pH 1.2). Four decolorized leaves per plant were examined and the presence of cell death was observed visually.

For the evaluation of drought stress sensitivity, *loh2* and *atr7* plants were germinated on soil in standard greenhouse conditions. Water supply was stopped 2 weeks after germination and the results were recorded after 2 more weeks. Relative water content (RWC) was measured using the formula RWC (%) = [(FM − DM)/(TM − DM)] × 100, where FM, DM, and TM are the fresh, dry, and turgid masses of the leaves, respectively. DM was determined after drying the leaves at 80 °C for 48 h and TM was measured after immersing the leaves in H_2_O for 24 h. Electrolyte leakage, which is an indicator of cell damage, was evaluated by measuring the increase in conductivity with an HI 873 conductivity meter (Hanna Instruments, Woonsocket, RI, USA). Leaves from *loh2* and *atr7* plants grown at optimal conditions and under drought stress were briefly washed with ultrapure water (conductivity of 1 µS). The leaves were then incubated in ultrapure water for 10 min. The conductivity of the resultant solution was measured and compared with the total conductivity obtained after boiling the leaves.

### DAB staining to detect hydrogen peroxide

The accumulation of H_2_O_2_ in plant tissues was visualized by histochemical detection, using DAB staining. To summarize, PQ-treated and control, *loh2* and *atr7* leaves were submerged in staining solution (1 mg/ml DAB (3, 3′-diaminobenzidine) in 0.05 M Tris acetate (pH 5)) in aluminium foil-wrapped tubes, followed by vacuum infiltration—two times for 10 min each at 25–100 mbar. Infiltrated samples were incubated in the dark overnight at room temperature. De-staining was performed by boiling in 96% ethanol for 5 min, discarding and replacing with fresh ethanol until no more color was leaching (3–4 times). One decolorized leaf per plant (× 4 plants) was examined and the presence of DAB staining was observed visually and photographed.

### Genetic mapping and cloning of *ATR7*

As *atr7* and its genetic background *loh2* are in *A. thaliana* ecotype Wassilewskija (Ws), *atr7* plants were crossed with *A. thaliana* ecotype Col-0 to generate a mapping population. F_1_ seedlings were also grown on MS media supplemented with 1.5 µM PQ to check if *atr7* is dominant or recessive. Thereafter, an F_2_ population generated from a cross between Col-0 and *atr7* was germinated on MS media supplemented with 1.5 µM PQ for 7–12 days to select PQ-tolerant individuals for genetic mapping. The selected plants were transferred to MS media without PQ for a few days before transferring them to pots containing soil. After DNA extraction, *atr7* was mapped roughly on chromosome 5 using SSLP (Simple Sequence Length Polymorphism) markers from the TAIR database (The *Arabidopsis* Information Resource; www.arabidopsis.org) (Supplementary Table 6). Later, a larger F_2_ population of 604 PQ-tolerant mutant plants was used for fine mapping with SSLP, InDel (Insertion/Deletion) and SNP (Single-Nucleotide Polymorphism) markers (Supplementary Table 7). Potential SNPs were selected by randomly sequencing a 1-kb region of *loh2* containing the two mutations. The SNP markers were designed using the Web SNAPER program.

After fine mapping of the *atr7* mutation in a region of approximately 100 kb on chromosome 5, the *atr7* and *loh2* plants were sequenced to find SNPs in that region. The FLORACLEAN™ Plant DNA Isolation kit (MP Biomedicals, CA, USA) was used to obtain nuclear DNA with minimal chloroplast or mitochondrial DNA contamination. Genomic DNA from each of the mutants was sequenced using Illumina HiSeq 2000 (Illumina Inc., CA, USA) at the University Medical Center Groningen (UMCG), according to the manufacturer’s protocol. Sequence contigs of ~ 200 bp obtained from *atr7* and *loh2* were then separately aligned to the Col-0 ecotype reference genome sequence (GenBank accessions: Chromosome 1, NC_003070, Chromosome 2, NC_003071, Chromosome 3, NC_003074, Chromosome 4 NC_003075, and chromosome 5, NC_003076). SNP list was generated with the help of CLC-Bio software (Qiagen, Hilden, Germany).

### Isolation of homozygous *atr7* knockout plants, construction of plasmids for complementation analysis, RNAi and overexpression lines, and plant transformation

T-DNA knockouts of the *ATR7* gene (locus *AT5G21280*) were identified from the available lines in the TAIR database (http://www.arabidopsis.org/). One line (KO, SALK_006796) with a T-DNA insertion in the first exon of *AT5G21280*, close to the nonsense mutation of *atr7*, was selected. Isolation of homozygous plants was done by genotyping using primers specific for the *ATR7* gene flanking the position of the insert and a primer recognizing the left border of the T-DNA (Supplementary Table 8).

To complement the *atr7* mutation with a functional *ATR7* gene and restore the PQ-sensitive phenotype, the full-length *ATR7* gene including both the promoter and 5´-UTR region was amplified from *loh2* genomic DNA using TaKaRa Taq™ Polymerase and primers PrRuG3477 and PrRuG3479 (Supplementary Table 8). The amplified product was first cloned into pGEM-T Easy vector (Promega, WI, USA) and then subsequently cloned into the plant expression vector pGreen II 0229 [30]. The integrity of the construct was confirmed by restriction digestion and sequencing. Subsequently, Agrobacterium-mediated gene transfer into *atr7* was performed and transgenics were selected using the herbicide Basta [31].

To generate *ATR7* RNAi lines, a partial coding region of *AT5G21280* was amplified using primers PrRuG3485 and PrRuG3486 (Supplementary Table 8) and cloned into the RNAi vector pFGC5941 (GenBank Accession No. AY310901; Arabidopsis Biological Resource Center stock number CD3-447). The amplified product was cloned in forward orientation using “inner” restriction enzyme sites *Asc*I/*Swa*I and in reverse orientation using “outer” restriction enzyme sites *Bam*HI/*Xba*I into plasmid pFGC5941. Thus, two reverse sequences of partial coding region of *AT5G21280* are separated by a *CHSA* intron spacer in the pFGC5941 construct. The integrity of the construct was confirmed by restriction digestion and sequencing. The RNAi construct was transformed into both *loh2* and *A. thaliana* ecotype Wassilewskija via Agrobacterium-mediated transfer. Transgenic plants were selected using the herbicide Basta [31].

### Analysis of ATR7 subcellular localization using GFP fusion

The full-length *ATR7* coding sequence was amplified without its stop codon from Arabidopsis Col-0 cDNA and the PCR product was cloned into vector pENTR/D–TOPO using pENTR Directional TOPO Cloning kit (Invitrogen, CA, USA). The sequence-verified entry clone was then transferred to vector pK7FWG2.0 using GATEWAY cloning (LR recombination reaction, Invitrogen, CA, USA). The resulting *Prom35S:ATR7*-*GFP* construct was verified by sequencing and introduced into *Arabidopsis thaliana* ecotype Col-0 plants either by transfecting mesophyll cell protoplasts or by Agrobacterium-mediated transformation using the floral dip method. For the transfection of mesophyll protoplasts, 40 µg plasmid DNA (*Prom35S:ATR7*-*GFP*) was added to 200 µL of the protoplasts suspension and incubated with the same volume of PEG solution (40% PEG 3500, 0.2 M mannitol, 0.1 M CaCl_2_). After transfection, the samples were diluted with 3 mL of W5 solution (154 mM NaCl, 125 mM CaCl_2_, 5 mM KCl, 2 mM MES, pH 5.7), collected by centrifugation at 100 g for 1 min, re-suspended in 1 mL W5 solution, and incubated in dark for 8–12 h in growth chamber before visualization. Agrobacterium-transformed transgenic plants were selected on MS media supplemented with 50 mg L^−1^ kanamycin. The presence of GFP signal was observed using a Leica TCS SP5 confocal laser scanning microscope. Nuclei were visualized with 4′,6-diamidino-2-phenylindole (DAPI) staining. Briefly, leaves were vacuum infiltrated with 1 µgmL^−1^ DAPI for 30 min. After rinsing in water, they were immediately used for microscopic analysis using a Leica TCS SP5 confocal microscope. DAPI was excited using the 405 nm laser and emission was collected between 440 and 470 nm.

### RNA isolation and qRT-PCR analysis of *ATR7* gene expression under environmental stresses

Seedlings from 1-week-old *loh2* plants were subjected to different abiotic stresses (heat at 45 °C for 1 h, cold at 1 °C for 2 h, growth on 150 mM NaCl, 250 μM CdCl_2_, 200 mM mannitol, and 10 mM H_2_O_2_) and RNA was extracted with Trizol reagent (Invitrogen) according to the manufacturer’s recommendations. Ten micrograms of total RNA was treated with DNA-free™ Kit (Ambion) to remove eventual DNA contamination. RNA integrity was checked on 1% (w/v) agarose gel and concentration measured with a Nanodrop ND-2000 spectrophotometer before and after DNase I digestion. Additionally, the quality and integrity of the RNA samples were analyzed on an RNA 6000 Lab-on-a-Chip using the Bioanalyzer 2100 (Agilent Technologies, Santa Clara, CA, USA). cDNA was synthesized from 2 µg of total RNA using RevertAid™ First Strand cDNA Synthesis Kit (Fermentas) with oligo-dT primers, according to the manufacturer’s instructions.

Quantitative real-time PCR (qRT-PCR) analysis was performed using an ABI PRISM 7900 HT PCR instrument (Applied Biosystems, Darmstadt, Germany). The following primers, designed using Primer3 software, were used for the qRT-PCR analysis of *ATR7* gene expression under abiotic and oxidative stresses: GTGGTGACGTCAGCTTGG (*ATR7* forward) and AAGGAAATTCCATGACGTCAC (*ATR7* reverse). For the analysis of *ATR7* gene expression in the *ATR7 KO* mutant, the following primers were used: CAAGCAAGAACCACGCGTCT (forward) and GCGACGAGCTTCAGCCATGT (reverse). All reactions contained 10 μL of SYBR Green Master Mix (Applied Biosystems), 25 ng of cDNA, and 200 nM of each gene-specific primer in a final volume of 20 μL. The qRT-PCRs were executed using the following program: 50 °C for 2 min, 95 °C for 10 min, followed by 40 cycles of 95 °C for 15 s and 60 °C for 1 min. Relative mRNA abundance was calculated using the comparative 2^−ΔΔCt^ method and normalized to the corresponding reference gene levels [32].

### Transcriptional profiling by RNA sequencing

For RNA-seq transcriptional profiling, total RNA was isolated from *loh2* and *atr7* seedlings grown on MS media or MS media supplemented with PQ using NucleoSpin^®^ RNA Plant kit of Machery-Nagel (Germany) according to the manufacturer’s protocol (http://www.mn-net.com/tabid/1327/default.aspx). DNase treatment to eliminate DNA contamination is included in the protocol. The concentration of the samples was analyzed with a NanoDrop ND-2000 spectrophotometer (Thermo Fisher Scientific, MA, USA). The quality and integrity of the RNA samples were analyzed on an RNA 6000 Lab-on-a-Chip using the Bioanalyzer 2100 (Agilent Technologies, CA, USA). Sample quality met the requirements for sample preparation. Illumina mRNA-Seq Sample Prep Kits were used to process the samples according to the Illumina protocol ´Preparing Samples for Sequencing of mRNA´ (1,004,898 Rev. D). Briefly, mRNA was isolated from the total RNA using poly-dT-oligo-attached magnetic beads. After fragmentation of the mRNA, cDNA synthesis was performed. The cDNA was used for ligation of the sequencing adapters and subsequent PCR amplification. The quality and yield after sample preparation were measured with a DNA 1000 Lab-on-a-Chip. The size of the resulting products was consistent with the expected size distribution (a broad peak of approximately 200-500 bp).

Sequencing using the Illumina HiSeq 2000 was performed (51 bp single end reads) according to manufacturer’s protocols at ServiceXS (Leiden, The Netherlands). A total of 4.5 pmol of DNA was used. Image analysis, base calling, and quality check were performed with the Illumina data analysis pipeline RTA v1.13.48 and/or OLB v1.9 and CASAVA v1.8.2.

### Bioinformatics analysis

Quality of obtained read sequences was tested with FastQC (http://www.bioinformatics.babraham.ac.uk/projects/fastqc). As no overrepresented sequences were detected, an additional 3′-trimming step was skipped.

Quantification was done using kallisto (v.0.43.0; bootstraps: 100) [33] against cDNA sequences (*Arabidopsis thaliana*: Araport11) [34]. Differential expression analysis was carried out using EdgeR package in R/Bioconductor [35]. FDR cutoff of ≤ 0.05 and log2 fold change ≥ 1 were used to identify significantly differentially expressed genes. Heatmaps and clustering for selected groups of genes were made using pheatmap R-package [36]. Significantly enriched GO terms were identified using GOSeq package [37] in R/Bioconductor with FDR cutoff of ≤ 0.05. Pathway analysis was done using MapMan (v 3.5.1R2) [38]. Co-expressed genes were identified using the CoNekT platform [39]. The multiple sequence alignment of protein sequences, obtained from Phytozome, was established using MUSCLE and the phylogenetic tree was constructed using Simple Phylogeny [40].

### Metabolome analysis of primary and secondary metabolites

Primary metabolites were determined by a previously established GC–MS protocol [41], using seedlings as plant material and six biological replicates. Chromatograms and mass spectra were evaluated by Chroma TOF^®^ 4.2 (Leco, MI, USA) and TagFinder 4.0 for the quantification and annotation of the peaks using the MPI Golm Metabolome Database (GMD, http://gmd.mpimp-golm.mpg.de/) [42]. The parameters used for the identification of the metabolites [43] are summarized in Supplementary Table 5. The amounts of metabolites were analyzed as relative metabolite abundances calculated by normalization of signal intensity to that of ribitol, which was added as an internal standard, and then by the fresh weight of the material. The whole dataset is provided in Supplementary Table 5. Data analysis was done using MetaboAnalyst 2.0 (www.metaboanalyst.ca) [44, 45].

### Electronic supplementary material

Below is the link to the electronic supplementary material.

Supplementary material 1 (JPEG 42 kb)

Supplementary material 2 (JPEG 198 kb)

Supplementary material 3 (JPEG 13 kb)

Supplementary material 4 (JPEG 295 kb)

Supplementary material 5 (JPEG 24 kb)

Supplementary material 6 (JPEG 37 kb)

Supplementary material 7 (JPEG 153 kb)

Supplementary material 8 (JPEG 25 kb)

Supplementary material 9 (JPEG 102 kb)

Supplementary material 10 (PDF 106 kb)

Supplementary material 11 (XLSX 3750 kb)

Supplementary material 12 (PDF 190 kb)

Supplementary material 13 (XLSX 17 kb)

Supplementary material 14 (XLSX 35 kb)

Supplementary material 15 (PDF 74 kb)

Supplementary material 16 (PDF 145 kb)

Supplementary material 17 (PDF 73 kb)

## Data Availability

Sequencing data is available at NCBI-SRA under BioProject ID: PRJNA475098.
