# Supplementary figures and images for: A novel seed plants gene regulates oxidative stress tolerance in Arabidopsis thaliana
Source: Cell Mol Life Sci. 2019 Jun 27;77(4):705–18. doi: 10.1007/s00018-019-03202-5 (PMC7040063; doi:10.1007/s00018-019-03202-5)

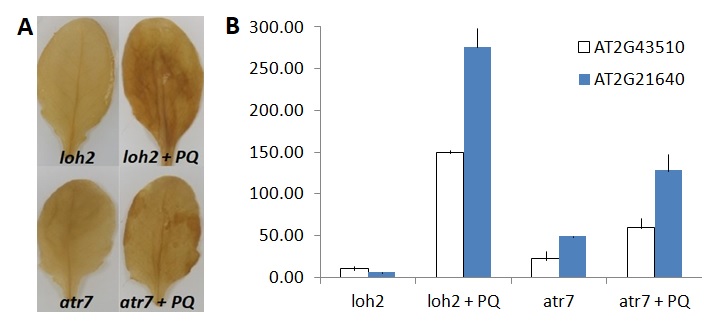

Supplement: Supplementary file 1 — Supplementary material 1 (JPEG 42 kb) [file 18_2019_3202_MOESM1_ESM.jpg]

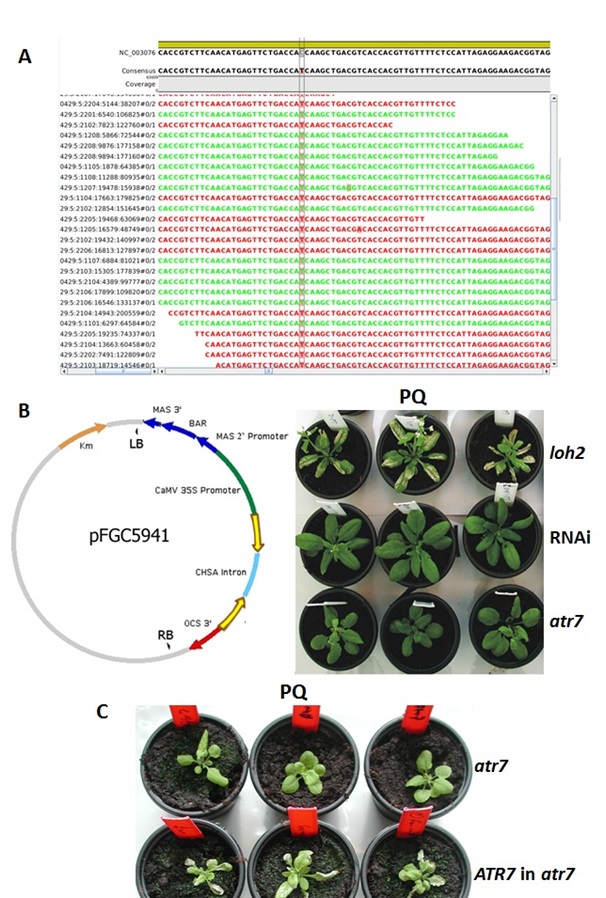

Supplement: Supplementary file 2 — Supplementary material 2 (JPEG 198 kb) [file 18_2019_3202_MOESM2_ESM.jpg]

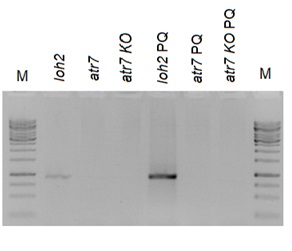

Supplement: Supplementary file 3 — Supplementary material 3 (JPEG 13 kb) [file 18_2019_3202_MOESM3_ESM.jpg]

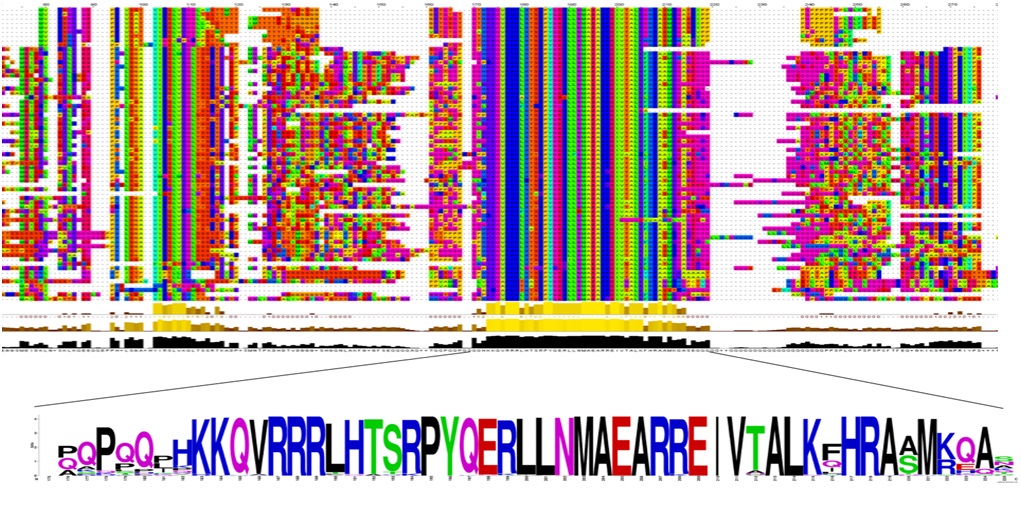

Supplement: Supplementary file 4 — Supplementary material 4 (JPEG 295 kb) [file 18_2019_3202_MOESM4_ESM.jpg]

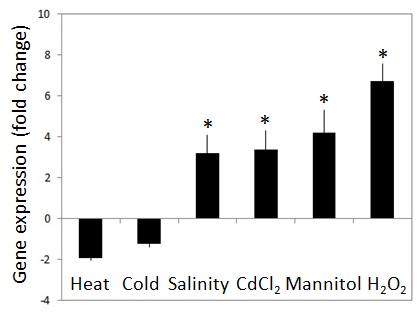

Supplement: Supplementary file 5 — Supplementary material 5 (JPEG 24 kb) [file 18_2019_3202_MOESM5_ESM.jpg]

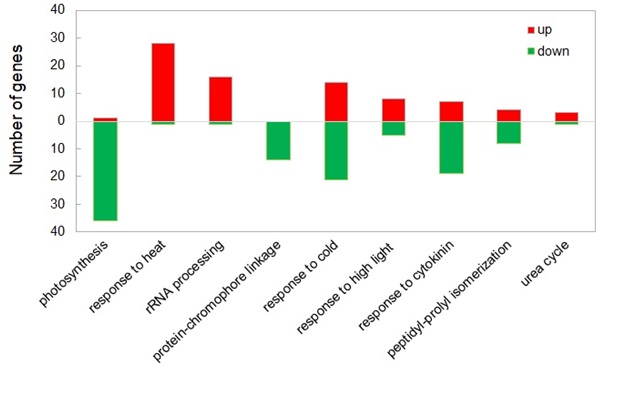

Supplement: Supplementary file 6 — Supplementary material 6 (JPEG 37 kb) [file 18_2019_3202_MOESM6_ESM.jpg]

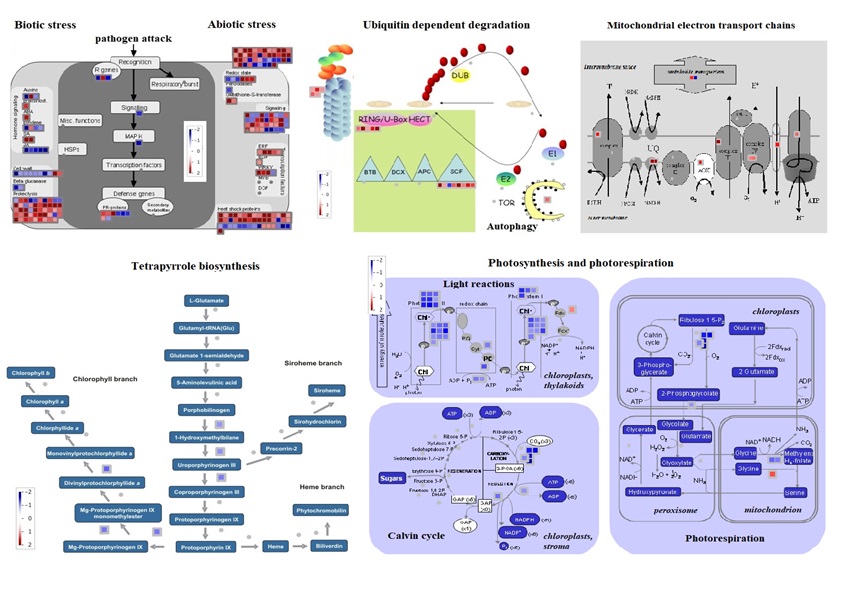

Supplement: Supplementary file 7 — Supplementary material 7 (JPEG 153 kb) [file 18_2019_3202_MOESM7_ESM.jpg]

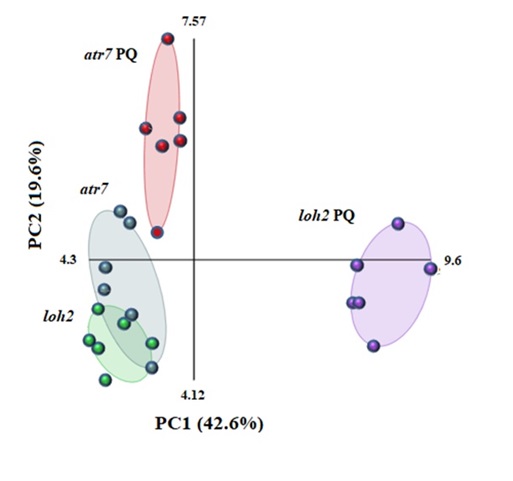

Supplement: Supplementary file 8 — Supplementary material 8 (JPEG 25 kb) [file 18_2019_3202_MOESM8_ESM.jpg]

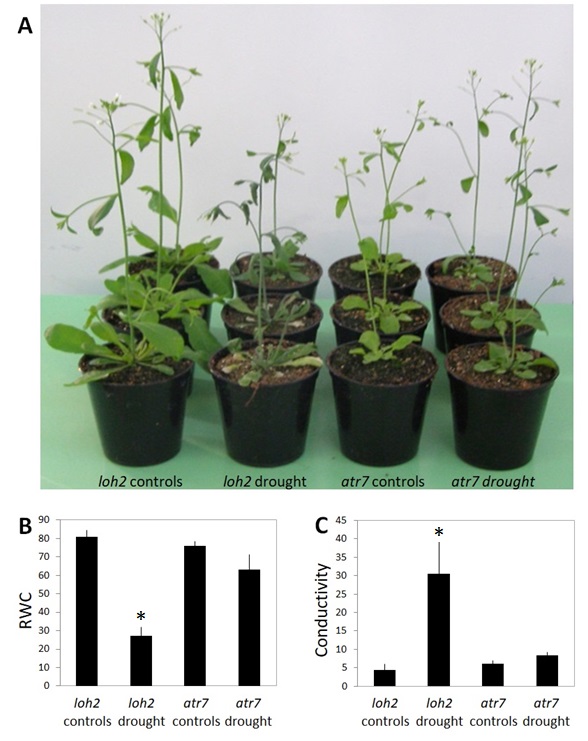

Supplement: Supplementary file 9 — Supplementary material 9 (JPEG 102 kb) [file 18_2019_3202_MOESM9_ESM.jpg]
