# Supplementary material for: A novel seed plants gene regulates oxidative stress tolerance in Arabidopsis thaliana
Source: Cell Mol Life Sci. 2019 Jun 27;77(4):705–18. doi: 10.1007/s00018-019-03202-5 (PMC7040063; doi:10.1007/s00018-019-03202-5)
Supplement: Supplementary file 10 — Supplementary material 10 (PDF 106 kb) [file 18_2019_3202_MOESM10_ESM.pdf]

Supplementary Table 1. ***ATR7*** **homologs in sequenced genomes available in Phytozome.**

**Species**	**ID**	**Phytozome ID**
A. coerulea	A. coerulea 1	Aqcoe7G430800.1.p
A. coerulea	A. coerulea 2	Aqcoe1G393700.1.p
A. coerulea	A. coerulea 3	Aqcoe1G393700.2.p
A. halleri	A. halleri	Araha.28327s0003.1.p
A. lyrata	A. lyrata	AL6G32940.t1
A. trichopoda	A. trichopoda	evm_27.model.AmTr_v1.0_scaffold00007.345
B. distachyon	B. distachyon 1	Bradi1g60291.2.p
B. distachyon	B. distachyon 2	Bradi1g60291.1.p
B. oleracea capitata	B. oleracea capitata 1	Bol025798
B. oleracea capitata	B. oleracea capitata 2	Bol035741
B. oleracea capitata	B. oleracea capitata 3	Bol036067
B. rapa FPsc	B. rapa FPsc 1	Brara.B00944.1.p
B. rapa FPsc	B. rapa FPsc 2	Brara.J01500.1.p
B. rapa FPsc	B. rapa FPsc 3	Brara.C00978.1.p
B. stacei	B. stacei	Brast02G208300.1.p
B. stricta	B. stricta	Bostr.26527s0080.1.p
C. clementina	C. clementina	Ciclev10008490m
C. grandiflora	C. grandiflora	Cagra.0056s0008.1.p
C. papaya	C. papaya	evm.model.supercontig_82.19
C. rubella	C. rubella	Carubv10003239m
C. sativus	C. sativus	Cucsa.055900.1
C. sinensis	C. sinensis	orange1.1g046333m
D. carota	D. carota	DCAR_001248
E. grandis	E. grandis	Eucgr.C00749.1.p
E. salsugineum	E. salsugineum	Thhalv10014216m
F. vesca	F. vesca	mrna08684.1-v1.0-hybrid
G. max	G. max 1	Glyma.04G057900.1.p
G. max	G. max 2	Glyma.06G058600.1.p
G. raimondii	G. raimondii 1	Gorai.010G094100.1
G. raimondii	G. raimondii 2	Gorai.009G039700.1
K. fedtschenkoi	K. fedtschenkoi	Kaladp0048s0010.1.p
K. laxiflora	K. laxiflora 1	Kalax.0201s0024.1.p
K. laxiflora	K. laxiflora 2	Kalax.0081s0008.1.p
K. laxiflora	K. laxiflora 3	Kalax.0081s0008.2.p
K. laxiflora	K. laxiflora 4	Kalax.0081s0008.3.p
K. laxiflora	K. laxiflora 5	Kalax.0081s0008.4.p
L. usitatissimum	L. usitatissimum 1	Lus10009801
L. usitatissimum	L. usitatissimum 2	Lus10038083

M. acuminata	M. acuminata 1	GSMUA_Achr3P01980_001
M. acuminata	M. acuminata 2	GSMUA_Achr2P21820_001
M. acuminata	M. acuminata 3	GSMUA_Achr8P30510_001
M. esculenta	M. esculenta 1	Manes.16G087800.1.p
M. esculenta	M. esculenta 2	Manes.03G055700.1.p
M. guttatus	M. guttatus	Migut.A00332.1.p
M. truncatula	M. truncatula 1	Medtr3g105580.2
M. truncatula	M. truncatula 2	Medtr3g105580.1
M. truncatula	M. truncatula 3	Medtr1g014750.1
O. thomaeum	O. thomaeum	Oropetium_20150105_10575A
P. hallii	P. hallii 1	Pahal.B04701.1
P. hallii	P. hallii 2	Pahal.B00623.1
P. persica	P. persica	Prupe.1G401300.1.p
P. trichocarpa	P. trichocarpa 1	Potri.006G217900.1
P. trichocarpa	P. trichocarpa 2	Potri.006G217900.2
P. virgatum	P. virgatum 1	Pavir.Ia03106.1.p
P. virgatum	P. virgatum 2	Pavir.Ib01892.1.p
P. virgatum	P. virgatum 3	Pavir.Ba00415.1.p
P. virgatum	P. virgatum 4	Pavir.Bb03330.1.p
P. vulgaris	P. vulgaris 1	Phvul.009G084600.1.p
P. vulgaris	P. vulgaris 2	Phvul.001G044400.1.p
P. vulgaris	P. vulgaris 3	Phvul.001G044300.1.p
P. vulgaris	P. vulgaris 4	Phvul.001G044200.1.p
R. communis	R. communis	29739.m003652
S. bicolor	S. bicolor 1	Sobic.001G344300.1.p
S. bicolor	S. bicolor 2	Sobic.002G374500.1.p
S. italica	S. italica 1	Seita.9G371400.1.p
S. italica	S. italica 2	Seita.2G388800.1.p
S. lycopersicum	S. lycopersicum 1	Solyc09g014950.1.1
S. lycopersicum	S. lycopersicum 2	Solyc08g067270.2.1
S. polyrhiza	S. polyrhiza	Spipo0G0137000
S. purpurea	S. purpurea	SapurV1A.0057s0340.1.p
S. viridis	S. viridis 1	Sevir.2G399300.1.p
S. viridis	S. viridis 2	Sevir.9G377100.1.p
T. cacao	T. cacao	Thecc1EG037421t1
T. pratense	T. pratense 1	Tp57577_TGAC_v2_mRNA40174
T. pratense	T. pratense 2	Tp57577_TGAC_v2_mRNA22154
V. vinifera	V. vinifera	GSVIVT01035506001
Z. marina	Z. marina 1	Zosma11g01290.1
Z. marina	Z. marina 2	Zosma81g01020.1
Z. mays	Z. mays 1	GRMZM2G109120_P01
Z. mays	Z. mays 2	GRMZM2G472234_P01
