# Supplementary material for: A novel seed plants gene regulates oxidative stress tolerance in Arabidopsis thaliana
Source: Cell Mol Life Sci. 2019 Jun 27;77(4):705–18. doi: 10.1007/s00018-019-03202-5 (PMC7040063; doi:10.1007/s00018-019-03202-5)
Supplement: Supplementary file 12 — Supplementary material 12 (PDF 190 kb) [file 18_2019_3202_MOESM12_ESM.pdf]

Supplementary Table 3. The 200 most regulated genes in *atr7 versus* its genetic background *loh2*. Positive values indicate higher expression in *atr7* while negative values indicate higher expression in *loh2*. FC, fold change.

**Gene**	**ATG code**	**Description**	**Log_2FC**
AT3G02790	AT3G02790	Zinc finger (C2H2 type) family protein	12.48
RPS27AA	AT1G23410	Ribosomal protein S27a / Ubiquitin family protein	10.86
ATEXO70G1	AT4G31540	Exocyst subunit exo70 family protein G1	9.30
CHR34	AT2G21450	Chromatin remodeling 34	7.38
RPL30A	AT1G36240	Ribosomal protein L7Ae/L30e/S12e/Gadd45 family protein	7.10
AT1G30170	AT1G30170	Hypothetical protein (DUF295)	6.96
AT4G05380	AT4G05380	P-loop nucleoside triphosphate hydrolase superfamily	6.88
HSP23.6	AT4G25200	Mitochondrion-localized small heat shock protein 23.6	6.67
AT2G35580	AT2G35580	Serine protease inhibitor (SERPIN) family protein	6.65
AT5G59390	AT5G59390	XH/XS domain-containing protein	6.35
DTX3	AT2G04050	MATE efflux family protein	6.25
AT5G01080	AT5G01080	Beta-galactosidase related protein	5.79
HSP17.6, HSP17.6II	AT5G12020	17.6 kDa Class II heat shock protein	5.75
ATOFP9, OFP9	AT4G04030	Transcription repressor	5.70
AT1G21520	AT1G21520	Hypothetical protein	5.59
MRD1	AT1G53480	Mto 1 responding down 1	5.35
ANAC085	AT5G14490	NAC domain containing protein 85	5.27
AT2G03130	AT2G03130	Ribosomal protein L12/ ClpS family protein	5.23
NAC090	AT5G22380	NAC domain containing protein 90	5.15
AT3G29633	AT3G29633	Hypothetical protein	4.99
AT4G07965	AT4G07965	Hypothetical protein	4.88
AT4G08593	AT4G08593	Hypothetical protein	4.88
AT3G48835	AT3G48835	Polynucleotide adenylyltransferase domain/RNA recognition motif	4.86

AT5G41660	AT5G41660	Transmembrane protein	4.79
EGC2, PNP-A	AT2G18660	Plant natriuretic peptide A	4.76
AT4G12735	AT4G12735	Hypothetical protein	4.75
AT2G42065	AT2G42065	DnaJ domain protein	4.72
NDB4	AT2G20800	NAD(P)H dehydrogenase B4	4.43
AT2G19850	AT2G19850	Transcription repressor	4.43
AT1G21280	AT1G21280	Copia-like polyprotein/retrotransposon	4.32
ARCK1	AT4G11890	Protein kinase superfamily protein	4.32
AT4G10440	AT4G10440	S-adenosyl-L-methionine-dependent methyltransferases	4.31
AT1G49150	AT1G49150	Transmembrane protein	4.28
AT5G13210	AT5G13210	Uncharacterized conserved protein UCP015417, vWA	4.26
AT3G48840	AT3G48840	RNA-binding (RRM/RBD/RNP motifs) family protein	4.24
PUB21	AT5G37490	ARM repeat superfamily protein	4.23
AT2G43960	AT2G43960	SWAP (Suppressor-of-White-APricot)/surp domain-containing	4.21
AT1G17960	AT1G17960	Threonyl-tRNA synthetase	4.15
CML41	AT3G50770	Calmodulin-like 41	4.07
HSP17.4, HSP17.4A	AT3G46230	Heat shock protein 17.4	4.04
HSP70, MED37C	AT3G12580	Heat shock protein 70	3.91
AT5G53230	AT5G53230	Hypothetical protein (DUF295)	3.91
HSP17.6A, HSP17.7	AT5G12030	Heat shock protein 17.6A	3.86
AT1G52905	AT1G52905	Hypothetical protein	3.84
AT3G54730	AT3G54730	Transcription repressor	3.69
RH49	AT1G71370	DEA(D/H)-box RNA helicase family protein	3.69
NDB3	AT4G21490	NAD(P)H dehydrogenase B3	3.66
ANAC044	AT3G01600	NAC domain containing protein 44	3.65
AT5G09570	AT5G09570	Cox19-like CHCH family protein	3.64
AT3G61111	AT3G61111	Zinc-binding ribosomal protein family protein	3.62
AT3G58150	AT3G58150	Optic atrophy 3 protein (OPA3)	3.56
AT4G04260	AT4G04260	Bromo-adjacent homology (BAH) domain-containing protein	3.55
AT5G58610	AT5G58610	PHD finger transcription factor	3.54

NUCL2, NUC-L2	AT3G18610	Nucleolin like 2	3.47
AT4G25330	AT4G25330	SAWADEE protein	3.45
AT5G60250	AT5G60250	Zinc finger (C3HC4-type RING finger) family protein	3.42
AT1G27565	AT1G27565	Hypothetical protein	3.41
SAG13	AT2G29350	Senescence-associated gene 13	3.39
TIM17-1	AT1G20350	Translocase inner membrane subunit 17-1	3.38
AT2G03965	AT2G03965	Hypothetical protein	3.37
AT2G18193	AT2G18193	P-loop containing nucleoside triphosphate hydrolases	3.37
HSP23.5	AT5G51440	HSP20-like chaperones superfamily protein	3.36
ANAC036	AT2G17040	NAC domain containing protein 36	3.36
DREB19	AT2G38340	Integrase-type DNA-binding superfamily protein	3.33
AT3G10930	AT3G10930	Hypothetical protein	3.32
AT1G71000	AT1G71000	Chaperone DnaJ-domain superfamily protein	3.28
SRO3	AT1G70440	Similar to RCD one 3	3.26
RH55	AT1G71280	DEA(D/H)-box RNA helicase family protein	3.24
AtRLP38	AT3G23120	Receptor like protein 38	3.14
AT1G47265	AT1G47265	Hypothetical protein	3.11
AT2G21640	AT2G21640	Marker for oxidative stress response protein	3.07
AT2S2, SESA2	AT4G27150	Seed storage albumin 2	3.06
AT5G24640	AT5G24640	Hypothetical protein	3.06
ASK13, SK13	AT3G60010	SKP1-like 13	3.05
AT1G02470	AT1G02470	Polyketide cyclase/dehydrase and lipid transport	3.04
HSP90-1	AT5G52640	Heat shock-like protein	3.02
AT3G14700	AT3G14700	SART-1 family	2.97
ATL55, RING1	AT5G10380	RING/U-box superfamily protein	2.95
AT2G14070	AT2G14070	Wound-responsive protein-like protein	2.92
AT1G47980	AT1G47980	Desiccation-like protein	2.91
AT3G24780	AT3G24780	Uncharacterized conserved protein UCP015417, vWA	2.90
NUDT24	AT5G19470	Nudix hydrolase homolog 24	2.90
ABCC3	AT3G13080	Multidrug resistance-associated protein 3	2.87
AT3G28580	AT3G28580	P-loop containing nucleoside triphosphate hydrolase	2.86
WRKY46	AT2G46400	WRKY DNA-binding protein 46	2.85

AT2G41730	AT2G41730	Calcium-binding site protein	2.84
AT3G45730	AT3G45730	Hypothetical protein	2.83
EIF6-1	AT2G39820	Translation initiation factor IF6	2.83
GMI1	AT5G24280	Gamma-irradiation and mitomycin c induced 1	2.82
AT3G24542	AT3G24542	Beta-galactosidase related protein	2.82
HSP17.8	AT1G07400	HSP20-like chaperones superfamily protein	2.81
CYP89A9	AT3G03470	Cytochrome P450, family 87, subfamily A, polypeptide 9	2.79
AT5G53240	AT5G53240	Hypothetical protein (DUF295)	2.73
DTX1	AT2G04040	MATE efflux family protein	2.73
AT2G04066	AT2G04066	MATE efflux family protein	2.69
CML30	AT3G29000	Calcium-binding EF-hand family protein	2.64
DOGT1, UGT73C5	AT2G36800	DON-glucosyltransferase 1	2.63
AT3G58270	AT3G58270	Phospholipase-like protein (PEARLI 4) with TRAF-like domain	2.56
CML47	AT3G47480	Calcium-binding EF-hand family protein	2.56
FKBP65	AT5G48570	FKBP-type peptidyl-prolyl cis-trans isomerase family protein	2.55
HSP17.4B	AT1G54050	HSP20-like chaperones superfamily protein	2.55
AT5G41763	AT5G41763	Hypothetical protein	2.54
BPC7	AT2G35550	Basic pentacysteine 7	2.51
AT4G19520	AT4G19520	Disease resistance protein (TIR-NBS-LRR class) family	2.47
AT5G41765	AT5G41765	DNA-binding storekeeper protein-related transcriptional regulator	2.45
AT5G56810	AT5G56810	F-box/RNI-like/FBD-like domains-containing protein	2.45
PER1	AT1G48130	1-cysteine peroxiredoxin 1	2.44
CRF6	AT3G61630	Cytokinin response factor 6	2.44
AT2G20720	AT2G20720	Pentatricopeptide repeat (PPR) superfamily protein	2.42
AT2G05580	AT2G05580	Glycine-rich protein family	2.41
HSFA2	AT2G26150	Heat shock transcription factor A2	2.39
HIPP13	AT5G52750	Heavy metal transport/detoxification superfamily protein	2.38
AT5G66052	AT5G66052	Transmembrane protein	2.37
UGT74E2	AT1G05680	Uridine diphosphate glycosyltransferase 74E2	2.35
BCS1, HSR4	AT3G50930	Cytochrome BC1 synthesis	2.34

AT1G35320	AT1G35320	Transmembrane protein	2.34
DTX17	AT1G73700	MATE efflux family protein	2.32
AT3G46845	AT3G46845	Hypothetical protein	2.30
UGT74E1	AT1G05675	UDP-Glycosyltransferase superfamily protein	2.27
GSTF7	AT1G02920	Glutathione S-transferase 7	2.26
AT3G28570	AT3G28570	P-loop containing nucleoside triphosphate hydrolases superfamily	2.26
AT1G78190	AT1G78190	Trm112p-like protein	2.25
RPP2E	AT5G40040	60S acidic ribosomal protein family	2.25
PBS3	AT5G13320	Auxin-responsive GH3 family protein	2.24
AT5G52930	AT5G52930	Hypothetical protein (DUF295)	2.24
RHL41, ZAT12	AT5G59820	C2H2-type zinc finger family protein	2.24
AT3G33528	AT3G33528	Hypothetical protein	2.24
UGT73C6	AT2G36790	UDP-glucosyl transferase 73C6	2.23
AT5G13830	AT5G13830	FtsJ-like methyltransferase family protein	2.23
WRKY50	AT5G26170	WRKY DNA-binding protein 50	2.23
HSP17.6C	AT1G53540	HSP20-like chaperones superfamily protein	2.19
AT3G15357	AT3G15357	Phosphopantothenoylcysteine decarboxylase subunit	2.16
QCR7-2	AT5G25450	Cytochrome bd ubiquinol oxidase, 14kDa subunit	2.16
FER2	AT3G11050	Ferritin 2	2.15
AT1G30160	AT1G30160	Hypothetical protein (DUF295)	2.13
SIB1	AT3G56710	Sigma factor binding protein 1	2.13
NUDT21	AT1G73540	Nudix hydrolase homolog 21	2.12
STZ, ZAT10	AT1G27730	Salt tolerance zinc finger	2.11
AT1G43910	AT1G43910	P-loop containing nucleoside triphosphate hydrolases superfamily	2.09
ANAC087	AT5G18270	NAC domain containing protein 87	2.08
AT4G37030	AT4G37030	Membrane protein	2.08
BGLU19	AT3G21370	Beta-glucosidase 19	2.08
AT1G73066	AT1G73066	Leucine-rich repeat family protein	2.05
MGD2	AT5G20410	Monogalactosyldiacylglycerol synthase 2	-2.04
AT4	AT5G03545	Expressed in response to phosphate starvation protein	-2.06
AT5G20790	AT5G20790	Transmembrane protein	-2.08

RPS19	ATCG00820	Ribosomal protein S19	-2.09
AT5G16350	AT5G16350	O-acyltransferase (WSD1-like) family protein	-2.09
AT2G30766	AT2G30766	Hypothetical protein	-2.12
LOX2	AT3G45140	Lipoxygenase 2	-2.12
AT5G05250	AT5G05250	Hypothetical protein	-2.13
GGCT2;1	AT5G26220	ChaC-like family protein	-2.14
ProT3	AT2G36590	Proline transporter 3	-2.18
IAA34	AT1G15050	Indole-3-acetic acid inducible 34	-2.22
ATCHX5	AT1G08150	Cation/hydrogen exchanger family protein	-2.24
PS2	AT1G73010	Inorganic pyrophosphatase 1	-2.26
AT1G29110	AT1G29110	Cysteine proteinases superfamily protein	-2.28
AT5G44850	AT5G44850	Protein with RNI-like/FBD-like domain	-2.29
RPL14	ATCG00780	Ribosomal protein L14	-2.30
RBCS3B	AT5G38410	Ribulose bisphosphate carboxylase (small chain) family protein	-2.30
ABCB13	AT1G27940	P-glycoprotein 13	-2.33
bHLH38, ORG2	AT3G56970	Basic helix-loop-helix (bHLH) DNA-binding superfamily protein	-2.35
DTX28	AT5G44050	MATE efflux family protein	-2.37
MTPC3	AT3G58060	Cation efflux family protein	-2.39
PPT2	AT3G01550	Phosphoenolpyruvate (pep)/phosphate translocator 2	-2.39
CYP96A9	AT4G39480	Cytochrome P450, family 96, subfamily A, polypeptide 9	-2.41
CORI3	AT4G23600	Tyrosine transaminase family protein	-2.41
AT1G48660	AT1G48660	Auxin-responsive GH3 family protein	-2.42
AT2G03020	AT2G03020	Heat shock protein HSP20/alpha crystallin family	-2.48
AT3G28220	AT3G28220	TRAF-like family protein	-2.48
RPL22	ATCG00810	Ribosomal protein L22	-2.50
CYP74B2, HPL1	AT4G15440	Hydroperoxide lyase 1	-2.54
AT5G47440	AT5G47440	Auxin canalization protein (DUF828)	-2.55
ARGAH2	AT4G08870	Arginase/deacetylase superfamily protein	-2.56
AT1G52100	AT1G52100	Mannose-binding lectin superfamily protein	-2.57
DTX50	AT5G52050	MATE efflux family protein	-2.60
AT1G01390	AT1G01390	UDP-Glycosyltransferase superfamily protein	-2.62

AT5G44260	AT5G44260	Zinc finger C-x8-C-x5-C-x3-H type family protein	-2.62
GER3	AT5G20630	Germin 3	-2.62
JAL23	AT2G39330	Jacalin-related lectin 23	-2.63
AT1G74820	AT1G74820	RmlC-like cupins superfamily protein	-2.70
MES18	AT5G58310	Methyl esterase 18	-2.84
ATCSLA15, CSLA15	AT4G13410	Nucleotide-diphospho-sugar transferases superfamily protein	-2.84
BGAL7	AT5G20710	Beta-galactosidase 7	-2.95
PEPC1	AT1G17710	Pyridoxal phosphate phosphatase-related protein	-2.96
GolS3	AT1G09350	Galactinol synthase 3	-2.97
AT1G23110	AT1G23110	Fold protein	-3.00
AT3G16670	AT3G16670	Pollen Ole e 1 allergen and extensin family protein	-3.03
LSU3	AT3G49570	Response to low sulfur 3	-3.07
AT2G47780	AT2G47780	Rubber elongation factor protein (REF)	-3.08
MLS	AT5G03860	Malate synthase	-3.11
ATSDI1	AT5G48850	Tetratricopeptide repeat (TPR)-like superfamily protein	-3.18
ICL	AT3G21720	Isocitrate lyase	-3.41
BGLU4	AT1G60090	Beta-glucosidase 4	-3.53
AT2G14247	AT2G14247	Expressed protein	-3.55
FOLB3	AT3G21730	Dihydroneopterin aldolase	-3.67
AT5G44440	AT5G44440	FAD-binding Berberine family protein	-5.00
IPS1	AT3G09922	Induced by phosphate starvation1	-5.99
ANS	AT2G38240	2-oxoglutarate (2OG) and Fe(II)-dependent oxygenase superfamily	-6.10
RALFL32	AT4G14010	Ralf-like 32	-9.93
