# Supplementary material for: A novel seed plants gene regulates oxidative stress tolerance in Arabidopsis thaliana
Source: Cell Mol Life Sci. 2019 Jun 27;77(4):705–18. doi: 10.1007/s00018-019-03202-5 (PMC7040063; doi:10.1007/s00018-019-03202-5)
Supplement: Supplementary file 15 — Supplementary material 15 (PDF 74 kb) [file 18_2019_3202_MOESM15_ESM.pdf]

Supplementary Table 6. List of SSLP, InDel (Insertion/Deletion) and SNP (Single-nucleotide polymorphism) markers used for the positional cloning of *ATR7*.

**Chromosome**	**DNA marker (TAIR)**	**Sequence (5´- 3´)**
1	NF21M12	TTACTTTTTGCCTCTTGTCATTG
		GGCTTTCTCGAAATCTGTCC
	NGA280	GGCTCCATAAAAAGTGCACC
		CTGATCTCACGGACAATAGTGC
2	lugsslp41	TGCATCAGTTTTGGTTGTGTGATCT
		GCTGTATTTTCCATAGGGGGCA
	NGA168	GAGGACATGTATAGGAGCCTCG
		TCGTCTACTGCACTGCCG
3	CIW11	GGCGAATTCTTAGACCATTATC
		AATAGTGTAAGGCACCTGTC
	CIW20	GAAGCCCTATCAAGCAACTC
		ATGGAGAATGAGACACTAGAC
4	JV32/33	GCCGCTCAACATGCTATAAA
		GTAGGTTCATCGACTGTATCTC
	CIW7	CTCCACTATGCAGTTGTCAG
		GTTGATGATAAGCACAAGGA
5	CA72	CCCAGTCTAACCACGACCAC
		AATCCCAGTAACCAAACACACA
	Lugsslp151	CGACATTGCTCTGTAATCTATCAC
		ACCAATTTCGACTGCTTCTACC
