# Supplementary material for: A novel seed plants gene regulates oxidative stress tolerance in Arabidopsis thaliana
Source: Cell Mol Life Sci. 2019 Jun 27;77(4):705–18. doi: 10.1007/s00018-019-03202-5 (PMC7040063; doi:10.1007/s00018-019-03202-5)
Supplement: Supplementary file 16 — Supplementary material 16 (PDF 145 kb) [file 18_2019_3202_MOESM16_ESM.pdf]

Supplementary Table 7. List of SSLP, InDel and SNP markers used for fine mapping of the *atr*7 mutation on chromosome 5.

**SSLP**	**DNA marker**	**Sequence (5´- 3´)**
1	CA72	CCCAGTCTAACCACGACCAC<br>AATCCCAGTAACCAAACACACA
2	NGA139	GGTTTCGTTTCACTATCCAGG<br>AGAGCTACCAGATCCGATGG
3	NGA106	GTTATGGAGTTTCTAGGGCACG<br>GAGAAGAACAAAAGGGGCA
**InDel**	**DNA marker**	**Sequence**
1	Marker 6 – 6564 kb	GGCAGAATGGTACGACTAACTC<br>CCTATCTCTTGTGTCGCACTCA
2	Indel 1-6921.1 kb	GCAACAGCCTGCCTGATATG<br>CTTCATCGGTAGCTTCTACAC
3	Indel 6 – 7428.5 kb	GTGACATTCCTAGACAATTAC<br>GTATCAGATAGAGAGGCCAGC
**SNP**	**DNA marker**	**Sequence**
1	BKN10249 - 5726.3 kb	GCCTTCCACAACGAACAATCC<br>GATCCAAAACCCTAGAAACACAAAC
2	BKN10389 - 6708 kb	ACGAAGTTTTCTACCTTTCTATTTACTGGT<br>TAACATGGATATTGACAGAGCCTGTTAGTC
3	BKN10406 – 6820.4 kb	GTAAGCATAAAGAGGCAGAAGTTGGTACCC<br>GACTGTGCCTACACATTCTAGTCAGATTGC
4	MASC9209 -7717.9 kb	AACAATTCCAACGTTAGAACGATCA<br>TCACTCGCGTAAATTGTTTTTATTT
5	Marker 2- 6323 kb	GATTCAGTTAGTTTTGGATAGAT<br>TGAGTAAGTGCTTGGTTGATTCTG
6	SNP2 – 7027.9 kb	TTAATCCCTACTTGCCTAAA<br>GCCTCTTCGCTGTTGACGGTT
7	SNP4 – 7205.9 kb	TAATCGTTGAATTTGATTTCT<br>GTTTTGAATTGAATCGATTGA
8	SNP 5 – 7337.6 kb	CTCGAGCATGTATCCCCATAA<br>AGCCAATGTGTCTGAGCTCCG
