# Supplementary material for: A novel seed plants gene regulates oxidative stress tolerance in Arabidopsis thaliana
Source: Cell Mol Life Sci. 2019 Jun 27;77(4):705–18. doi: 10.1007/s00018-019-03202-5 (PMC7040063; doi:10.1007/s00018-019-03202-5)
Supplement: Supplementary file 17 — Supplementary material 17 (PDF 73 kb) [file 18_2019_3202_MOESM17_ESM.pdf]

Supplementary Table 8. List of primers for genotyping, complementation analysis, RNAi lines, and subcellular localization using GFP fusion.

Experiments	Primer name	Sequence (5´ - 3´)
Genotyping of KO line SALK_006796		ATGGTCAGATCAAACAAGCAAG<br>AGCAAGCCAGTCGTCTCC
Intact gene in *atr*7	PrRuG3477<br>PrRuG3479	GGCTGTTAGCTAGGCATGAATCAAG<br>GACAACTCCTTTCTGAATGTATC
RNAi	PrRuG3485<br>PrRuG3486	AATCTAGA GGCGCGCCAGAACCACGCGTCTCTTC<br>*Xba*I *Asc*I<br>TTGGATCC ATTTAAATGAGGAGGCGGCGGAGAGA<br>*Swa*I *Bam*HI
Subcellular localization using GFP fusion		CACCATGGTCAGATCAAACAAGCAAG<br>AGCAAGCCAGTCGTCTCC
